# Mitigating Organophosphate Nerve Agent, Soman (GD)-Induced Long-Term Neurotoxicity: Saracatinib, a Src Tyrosine Kinase Inhibitor, as a Potential Countermeasure

**DOI:** 10.21203/rs.3.rs-6674766/v1

**Published:** 2025-06-24

**Authors:** Nyzil Massey, Suraj S Vasanthi, Claire Holtkamp, Christina Meyer, Nikhil S Rao, Luis G Gimenez-Lirola, Chong Wang, Hyunmook Im, Avinash S Bevoor, Sridhar Kannurpatti, Thimmasettappa Thippeswamy

**Affiliations:** Iowa State University; Iowa State University; University of Kentucky; Iowa State University; Iowa State University; Iowa State University; Iowa State University; Iowa State University; Iowa State University; New Jersey Medical School; Iowa State University

**Keywords:** Organophosphate nerve agents, Soman (GD), Saracatinib, Neurotoxicity, Neuroinflammation, Neurobehavior, Nitro-oxidative stress, Glial scar, Epilepsy

## Abstract

**Background:**

Acute exposure to soman (GD), an organophosphate nerve agent (OPNA), irreversibly inhibits acetylcholinesterase (AChE), induces seizures, and could be fatal if not treated immediately. Existing medical countermeasures (MCMs- atropine, oximes, and benzodiazepines) mitigate the acute life-threatening cholinergic symptoms but have limited protection against long-term neurological damage in survivors. This indicates a need for an effective adjunct therapy to mitigate cognitive, behavioral, and brain pathology associated with OPNA exposure. Saracatinib (SAR), a selective Src tyrosine kinase inhibitor, has emerged as a potential candidate, given its protective properties in experimental models of excitotoxicity and neuroinflammation. Here, we evaluate the therapeutic efficacy of SAR in mitigating long-term neurological deficits triggered by acute exposure to soman in a rat model.

**Methods:**

Mixed-sex adult Sprague Dawley rats were exposed to soman (132 μg/kg, s.c.) and immediately treated with atropine (2 mg/kg, i.m.) and HI-6 (125 mg/kg, i.m.). Seizure severity was quantified for an hour before administering midazolam (3 mg/kg, i.m.). One-hour post-midazolam, SAR/vehicle was administered orally and daily for 18 weeks in the diet. After behavioral testing, brain MRI, and EEG acquisition, animals were perfused with 4% paraformaldehyde at 18 weeks post-soman. Serum and CSF were collected for nitro-oxidative markers and proinflammatory cytokines. Brains were processed for neuroinflammation and neurodegeneration markers.

**Results:**

SAR treatment attenuated the soman-induced anxiety/fear-like behavior and motor impairment and modulated the severity, frequency, and duration of seizures. Despite improved hippocampal functional connectivity (MRI), SAR did not reverse soman-induced learning and memory deficits at 5–7 weeks. However, 18 weeks of SAR treatment demonstrated anti-inflammatory and antioxidant properties, mitigated reactive gliosis and neurodegeneration, and partially protected somatostatin parvalbumin inhibitory neurons. The glial scars in the amygdala were reduced in SAR-treated animals compared to the vehicle-treated group.

**Conclusions:**

Long-term SAR treatment revealed disease-modifying effects by protecting the brain from soman-induced neuroinflammation and neurodegeneration, while also reducing seizure severity and modulating the frequency and duration of seizures. Furthermore, it provided partial protection against behavioral impairments and MRI deficits in the short term. These findings highlight the therapeutic potential of Src tyrosine kinase inhibition in addressing chronic neurotoxicity induced by soman.

## Background

Organophosphate (OP) nerve agents (OPNAs), such as sarin, soman, tabun, and VX, were originally developed as insecticides but have since become notorious for their potential use as chemical warfare agents. Their extreme toxicity, relative ease of production, and demonstrated misuse as in the 1995 Tokyo subway sarin attack (Sugiyama et al. 2020), which underscores their persistent global threat. OPNAs excert their toxic effects primarily by irreversibly inhibiting acetylcholinesterase (AChE), resulting in an accumulation of acetylcholine and a subsequent cholinergic crisis. Clinically, acute OP exposure leads to debilitating muscarinic and nicotinic symptoms (e.g., bronchoconstriction, muscle fasciculations, and respiratory failure), and central nervous system effects such as anxiety, confusion, seizures, and, in severe cases, death. Although current medical countermeasures (MCMs), atropine, oximes (e.g., pralidoxime, HI-6), and benzodiazepines (diazepam, midazolam) can markedly reduce the acute mortality associated with OPNA intoxication, they do not prevent or reverse the long-term neurological outcomes if the treatment is delayed. Survivors of OPNA exposure often develop persistent cognitive, behavioral, and psychiatric impairments, including memory loss, and chronic anxiety or depression. These long-term deficits likely stem from excitotoxicity, oxidative stress, and neuroinflammation triggered during the acute cholinergic crisis, highlighting the need for therapies that target both immediate life-threatening symptoms and long-term neurological damage.

Beyond the standard of care using MCMs, research into new therapies, such as improved efficacy of oxime drugs and enzyme-based bioscavengers, show substantial promise in experimental models and early clinical trials. Enhanced oximes aim to penetrate the blood-brain barrier more effectively, which could improve outcomes by reactivating AChE directly in the central nervous system that current therapies achieve to a limited extent. Bioscavengers, on the other hand, bind and neutralize nerve agents before they cause further brain injury. While these discoveries have produced encouraging results in controlled settings in short-term studies, their efficacy and applicability for chronic neurotoxicity are unclear.

The Src Family tyrosine kinases (SFKs) regulate cell growth, adhesion, migration, differentiation, and immune responses. This family includes nine members Src, Fyn, Lyn, Yrk, Yes, Blk, Fgr, Hck, and Lck. Among them, Src and Fyn are relevant in the brain as they modulate synaptic plasticity, myelination, and oligodendrocyte formation. Notably, in response to seizures, SFKs phosphorylation and their interaction with other signaling molecules such as tau in the central nervous system can initiate neuronal hyperexcitability, reactive gliosis (neuroinflammation), and neurodegeneration. saracatinib (SAR), a selective SFK inhibitor (also known as AZD0530), treatment mitigated the long-term neurological damage induced by acute exposure to chemoconvulsants such as diisopropylfluorophosphate (DFP), an OPNA surrogate, and kainate (KA, a glutamate receptor agonist) models. Chemoconvulsant-induced *status epielpticus* (SE) leads to synaptic dysfunction, neuronal cell death, and cognitive impairment. Our previous studies in the rat KA model demonstrated the potential role for SAR in mitigating SE-induced effects (Sharma et al., 2021). In those studies, SAR treatment was limited for a week and the behavioral outcomes were not investigated. In the rat OPNA (DFP) model, the short-term SAR dosing regimen was not as effective as in the KA model. Therefore, we tested the efficacy of SAR in the diet, treated in decreasing doses, for long-term in the rat soman model. DFP is a surrogate of soman and shares common chemical and functional properties, such as induction of seizures upon acute exposure and long-lasting, irreversible brain injury. However, despite such similarities, soman is highly volatile than DFP and highly regulated.

In this study, SAR treatment in the diet reached a steady therapeutic concentrations in the serum and the decreasing concnetrations of the drug in the diet also correlated with the serum levels. Such concentrations mitigated the soman-induced anxiety-like behavior, improved motor function, and reduced both convulsive and non-convulsive seizure severity following soman exposure. SAR also reduced reactive gliosis and neurodegeneration, partially preserved parvalbumin and somatostatin inhibitory neurons, and restored hippocampal connectivity and anti-inflammatory/antioxidant responses. However, it failed to rescue recognition or contextual fear learning and memory deficits, alter epileptiform spiking, or affect glial scarring. Overall, these outcomes underscore the disease-modifying effects of SAR in the soman model of long-term neurotoxicity.

## Methods

### Animals source, care and, ethical considerations

Adult male and female Sprague Dawley rats (7–8 weeks old, weighing 250–300 g) were obtained from Charles River Laboratories (USA) for use in this study. Animals were housed individually in an enriched environment under controlled conditions with a 12-hour light/dark cycle. Both male and female rats were housed in the same room. Animals were allowed 3 days of acclimatization prior to the beginning of experiments. All experimental procedures complied with protocols approved by the Institutional Animal Care and Use Committee (IACUC) at Iowa State University (Protocol number: 23–114) and adhered to the NIH ARRIVE guidelines for the Care and Use of Laboratory Animals. Exposure to soman was conducted at MRI Global (Kansas City, MO), while all subsequent experimental activities occurred within the Laboratory Animal Resources facility at Iowa State University. At the conclusion of the 18-week study period, animals were humanely euthanized via intraperitoneal injection of pentobarbital sodium and phenytoin sodium (100 mg/kg) in accordance with the American Veterinary Medical Association’s (AVMA) Guidelines for Euthanasia.

### Chemicals and reagents

Soman (purity > 95%, confirmed by GC–MS) was administered to animals at MRI Global (Kansas City, MO, USA) following an approved IACUC protocol. The soman was diluted in cold phosphate-buffered saline (PBS). HI-6 (99.9% purity, validated by LC/MS; Kalexsyn, Kalamazoo, MI, USA) and atropine sulfate monohydrate (ATS, 99.9% purity, verified by LC–MS; Tokyo Chemical Industry, USA) were each diluted in saline to achieve final concentrations of 250 mg/ml and 5 mg/ml, respectively. All chemicals administered to animals underwent verification by LC-MS to ensure purity and identity. Midazolam (MDZ), prepared as a 5 mg/ml stock solution, was provided by MRI Global. SAR was generously provided by AstraZeneca via the Open Innovation Program and was diluted in 0.5% hydroxypropylmethylcellulose and 0.1% Tween 80 (VEH). The SAR solution was prepared by continuously stirred at room temperature to avoid precipitation as detailed in (Gage et al. 2021). The euthanasia solution, containing pentobarbital sodium and phenytoin sodium, was acquired from the Pharmacy at the ISU Lloyd Veterinary Medical Center. Detailed information regarding the assay kits utilized in this study is specified in the respective experimental sections.

### Soman exposure protocol, medical countermeasures, and animal allocation

**Figure 1:**
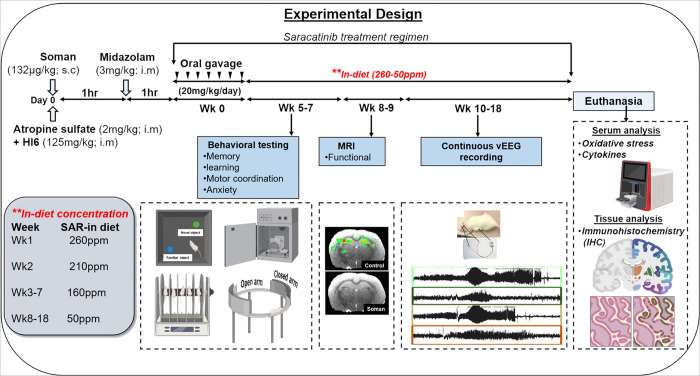
Experimental design. 18-week study with various treatments, analyses in live animals, and sample collection after euthanasia.

A total of 100 rats (50/sex) were used in this study. Male and female animals were randomly assigned to each treatment group. For animal treatment groups, treatment duration and allocated animal numbers refer to Table 1. Animals that were not exposed to soman either received cold phosphate-buffered saline (PBS, i.m.) or 0.5% hydroxypropyl methylcellulose (HPMC, oral gavage). Rest of soman-exposed animals (soman 1.2 × LD50, 132 μg/kg, s.c.) received received HI-6 (125 mg/kg, i.m.) and atropine sulfate (2 mg/kg, i.m.) within one minute of soman exposure to minimize peripheral cholinergic toxicity and reduce mortality rates. Additionally, midazolam (3 mg/kg, i.m.) was administered one hour post-exposure to control seizures and prevent mortality associated with severe SE. Soman exposed animals from both sexes with comparable SE severity were further randomly categorized into two groups (with or without SAR treatment). Aditionally, details regarding the specific animal numbers used for each analysis can be found in Table 2. Refer to Fig. 1 for the experimental design of the study.

### Assessment of behavioral seizures and status epilepticus (SE) severity

Behavioral seizure activity was closely observed and recorded for 60 minutes following soman exposure, continuing until the administration of midazolam. Two trained observers independently monitored each animal, scoring seizure severity in real-time using a standardized 1 to 5 scale. Seizure stages, characterized as follows, were assessed. Stage 1: Excessive salivation, lacrimation, urination, defecation, mastication, and chewing. Stage 2: Increased severity of stage 1 symptoms accompanied by tremors, wet-dog shakes, head nodding or neck jerks, kyphosis, and opisthotonos. Stage 3: Appearance of forelimb clonus, Straub tail response, rearing, and rigid limb extension. Stage 4: Marked by repeated rearing, severe forelimb clonus, continuous Straub tail, and sustained rigid extension. Stage 4: Severe generalized seizures characterized by loss of posture, forelimb clonus, rearing, falling, and convulsive episodes. Stage 5: Sustained generalized tonic-clonic seizures. Seizure stage severity was scored in real-time by two independent observers. The duration of convulsive seizures was recorded and assigned as a SE severity score prior to administering midazolam 60 minutes after exposure.

### SAR treatment regimen

Two animal groups, soman + SAR (soman-exposed) and control + SAR (without soman exposure) were orally gavaged bolus SAR dose at 20 mg/kg for first 7 days post soman. After receiving the oral SAR, the respective groups were given ad-libitum SAR mixed into their diet with gradually decreasing concentrations over 18 weeks, refer to Table 3. The SAR levels in the diets tapered from 260 to 50 ppm to reach a target daily dosage range of 30–2.5 mg/kg per day. The control diet (regular rat chow), with a similar composition and ingredients was procured from the same supplier and provided to both the control group and the soman exposed groups without SAR treatment. Blood samples were collected after each dietary change to compare serum SAR concentrations between the groups. Refer to Fig. 1 for the experimental design of the study.

### Surgical implantation of telemetry devices and video-EEG (vEEG) seizure quantification

To investigate the potential progression from soman-induced acute status epilepticus (SE) to chronic epilepsy, 32 rats (16/group, 8/sex) underwent telemetry device implantation for continuous EEG monitoring at Iowa State University. Prior to surgery, animals received analgesia with Buprenorphine (0.3 mg/kg, s.c.) and antibiotic prophylaxis with enrofloxacin (5 mg/kg, s.c.). Anesthesia induction was achieved using 3.0% isoflurane delivered at 1 L/min O₂ and subsequently maintained at 1.0–2.0% isoflurane throughout the procedure via inhalation. After surgical preparation, the parietal bones were exposed by making a midline incision and removing the underlying connective tissues. Small burr holes were drilled bilaterally to implant EEG electrodes on the dura mater over each cortical hemisphere. A telemetry transmitter was placed within a subcutaneous pocket in the flank region, and electrodes were fixed securely using dental cement (A-M Systems, Carlsborg, WA). Post-surgery, animals were housed individually on telemetry receiver plates (RPC-1; Data Sciences International, DSI), facilitating continuous synchronized video-EEG (vEEG) recording using Ponemah Software (DSI). Animals were continuously monitored to assess seizure frequency and severity, quantifying the progression from status epilepticus (SE) to chronic epilepsy. Refer to Fig. 1 for experimental design of the study.

### Quantification of spontaneously recurring seizures (SRS)

To quantify spontaneously recurring seizures (SRS), a seizure-free and epileptiform-free EEG segment was first identified for each animal to establish baseline parameters for subsequent SRS detection. EEG recordings were scrutinized for episodes characterized by high-frequency and/or high-amplitude epileptiform spikes, cross-referenced with simultaneous video recordings to ensure accurate seizure identification. Artifacts from electrical noise, grooming behaviors, and exploratory activities were carefully identified and excluded, consistent with procedures detailed in our previous studies. Seizure events detected automatically by NeuroScore software (version 3.4.0, DSI) were manually confirmed by analyzing synchronized video evidence and assessing corresponding changes in EEG power spectra.

### Novel object recognition (NOR) and open field tests

The novel object recognition (NOR) test is a behavioral test used to evaluate various aspects of memory and learning in rodents. The NOR is conducted over three days: habituation/open field test day, testing day, and probing day. Before the NOR, the rats were handled consistently to minimize stress or anxiety that could affect the results. On the habituation/open field test day (to assess anxiety-related behavior), the rat was given 10 minutes to explore the arena, which measures 40 × 40 inches, without any objects. On the testing day, two similar objects, as prescribed by Stoeling, were placed diagonally in the arena, and the rat was positioned in one corner to explore both objects for 5 minutes. After a 24-hour interval, on the probing day, one of the original objects was replaced with a completely new one, and the rat was again placed in the corner to explore the objects for 5 minutes. The rat’s movement was recorded using the ANY-maze software (Stoeling Co., USA). Time spent, number of entries, and distance travelled by the rat in the center, as well as the time spent with the novel versus the familiar object on the probing day, are analyzed across different groups and sexes. Discrimination index, DI = (t^novel^− t^familiar^)/(t^novel^ + t^familiar^) and recognition index, RI = t^novel^/total time exploration time were determined using these formulas.

### Elevated zero/circular maze test

The anxiety-like behaviors are assessed using an elevated zero/circular maze test. Unlike the open field test, the elevated circular platform (47-inch diameter) is positioned 20 inches above the ground. The platform, 4 inches in width, is divided into two enclosed arms, each with 12-inch high walls opposite one another and two open arms between the enclosed arms. Rats were placed at the junction of the open and closed arms and allowed to explore the maze for 5 minutes. The rat movements were recorded remotely with an overhead camera, using ANY-Maze software (Stoelting Co., USA). The time spent by the rats in the closed arms was quantified and analyzed across different groups and sexes.

### Accelerated rotarod test

Motor learning and coordination were assessed using an accelerating rotarod apparatus (AccuRotor 4-channel, Omnitech Electronics Inc., and Start Fusion v6.2 AccuRotor Edition Software). The acceleration rate ranged from 5 to 60 rpm, with a total trial duration of 180 seconds. The experiment was conducted over 3 days, consisting of 2 days of training and 1 day of testing, with a 24-hour rest period between each day. Motor coordination and learning were evaluated across three days, and the mean latency to fall (time spent on the rotating rod) on testing day was recorded and compared between groups and sexes.

### Contextual and cued fear conditioning test

The rats were tested for contextual and cued fear conditioning to evaluate their learning and memory by examining the association between a conditioned stimulus (a tone) and an unconditioned stimulus (an electric foot shock) The test consisted of two phases: conditioning (Day 1) and probing (Day 2). During the conditioning phase, rats were allowed to explore the apparatus for 2 minutes, followed by an auditory tone (70–80 dB) lasting 20 seconds. A 0.7-mA foot shock was then delivered continuously during the last 2 seconds of the tone. This conditioning procedure was repeated four times per session, with an 80 second inter-trial interval, followed by a rest period of 160 seconds. Twenty-four hours after the conditioning session, the rats underwent the probing phase, which followed the same procedure as the conditioning phase, except that no foot shock was administered. The movement of the rats was monitored using a camera and recorded via ANY-maze software (Stoelting Co., USA). The freezing time (at tones) during the conditioning and probing phases was analyzed, and compared across groups and sexes. The percentage change in freezing time (from conconditioning to probe) for all tones was compared using the formula (Freezing time^Conditioning^-Freezing time^Probe^)/Freezing time^Conditioning^.

### Functional MRI acquisition and image processing

Brain MRI measures in the rats were acquired using a GE Discovery MR901 7 Tesla horizontal bore scanner. Animals were induced initially with 3% isoflurane followed by intraperitoneal initial bolus of Dexmeditomedine 0.125mg/kg. Thereafter, animals were maintained with 0.5% isoflurane in 30% O_2_/70%N_2_ delivered through a nose cone throughout the MRI scan duration. Warm air circulated in the bore to keep body temperature stable and respiratory rate was monitored with a pneumatic pillow. Animals’ heads were fixed using bite bars to limit motion and placed in the bore of a volume transmit RF coil of a 7cm diameter with a four-channel surface receive coil. T2 steady-state contrast images were obtained using a fast imaging employing steady-state acquisition (FIESTA) sequence for rapid scanning and coverage of the whole brain. MRI parameters were: TR/TE = 4.3ms/1.9ms, 108 axial slices of thickness 0.3mm, and in-plane resolution of 0.13mm × 0.13mm covering a 2.5cm × 2.5cm field of view. Restingesting state fMRI data acquisition used a T2* weighted gradient-recalled echo (GRE) segmented echo planar imaging (EPI) sequence with blood oxygenation level-dependent (BOLD) contrast. The imaging parameters were, TR/TE = 1500ms/15ms, 4 segments per image, 100 repetitions, slice thickness of 0.9mm, field of view = 2cm × 2cm, 96 × 96 matrix, 22 slices covering the whole brain from the olfactory lobe to the cerebellum. All images were acquired in DICOM format and reconstructed using the Neuroimaging Informatics Technology Initiative (NITRC) program for preprocessing and registration, utilizing the BioImage Suite (Duncan et al. 2004). The sigma rat MRI template (Barrière et al. 2019) served as the reference spatial template for nonlinearly registering a single control rat brain MRI using the BioImage Suite. The resulting baseline template was used for the nonlinear registration of all other subject MRIs. Functional MRI data from all animals were linearly co-registered to the control study subject template and then motion-corrected using the fifth image of each respective animal, employing the Analysis of Functional NeuroImages (Cox 1996). The hippocampal resting-state functional connectivity (RSFC) networks for each animal were assessed, and the parameters were established as determined in our previous publication (Putra et al. 2024b).

### Blood, cerebrospinal fluid (CSF), and tissue collection

Blood was collected via cardiac puncture under terminal anesthesia. Animals were placed in a supine position, and the thoracic cavity was surgically opened. A sterile syringe equipped with a 21–25 gauge needle was inserted into the left ventricle through the chest wall to aspirate blood. Collected blood was centrifuged at 1000–1500 × g for 10 minutes to separate serum. Cerebrospinal fluid (CSF) collection was performed at the cisterna magna under terminal anesthesia. Animals were positioned with their heads angled downward at approximately 45° to facilitate CSF access. A depression located between the occipital protuberance and the wing of the atlas was identified, and a sterile needle attached to a syringe was inserted perpendicular to the skin surface directly into the cisterna magna without incision to prevent blood contamination. A noticeable change in resistance indicated proper needle placement within the cisterna magna. Clear CSF was slowly aspirated into the syringe. Collected serum and CSF samples were immediately aliquoted and stored at −80°C for future analyses. Brain tissue designated for gene expression studies was quickly snap-frozen in liquid nitrogen (LN2) and stored at −80°C. Refer to Fig. 1 for the experimental design of the study.

### Cytokine and chemokine analysis using Luminex assays

Cytokine and chemokine levels (TNF-α, IL-6, IL-1α, IL-18, IL-17A, and MCP-1) in serum and CSF samples were assessed using a customized MILLIPLEX^®^ Rat Cytokine/Chemokine kit (Millipore Sigma, RECYTMAG-65K-08C). For each assay, 25 μL of sample (serum or CSF) was mixed with 25 μL of primary antibodies conjugated to magnetic microspheres and incubated overnight at 4°C. After incubation, wells were washed three times, followed by a one-hour incubation with secondary antibodies. Subsequently, streptavidin/phycoerythrin was added to each well and incubated for 30 minutes following additional washing steps. All samples were analyzed in duplicate using a Bio-Plex plate reader (Bio-Rad). Cytokine concentrations were calculated based on standard curves established with known cytokine standards, with final concentrations expressed in pg/mL.

### Liquid chromatography-mass spectrometry (LC-MS/MS) analysis for SAR

LC separations were performed using an Agilent Technologies 1290 Infinity Binary Pump UHPLC system equipped with an Agilent Technologies Eclipse C18 analytical column (1.8 μm, 2.1 mm × 100 mm), coupled to an Agilent Technologies 6540 UHD Accurate-Mass Q-TOF mass spectrometer (Agilent Technologies, Santa Clara, CA). Samples (6 μL per injection, or 0.5 μL for food pellet samples) were introduced into the LC system. Chromatographic separation was conducted at 40°C, with a flow rate of 0.400 mL/min. The mobile phases consisted of solvent A (water containing 0.1% formic acid) and solvent B (acetonitrile containing 0.1% formic acid). Gradient conditions started at 1% B, increased linearly to 50% B over 7 minutes, then further to 100% B over 2 minutes, followed by a hold at 100% B for 6 minutes before returning to 1% B over 2 minutes. A post-run equilibration at 1% B was maintained for 5 minutes between analyses. SAR was detected using electrospray ionization in positive ion mode. Nitrogen served as the ion source drying gas at a flow rate of 12 L/minute and a temperature of 350°C, with a nebulizer pressure of 25 psi. The sheath gas flow rate was 11 L/minute at 400°C. Capillary and nozzle voltages were set to 4000 V and 1750 V, respectively. The mass spectrometer operated in high-resolution mode (4 GHz) with an MS scan range of m/z 100–1700 and an MS/MS range of m/z 60–350. Acquisition rates were 10 spectra/second for MS and 5 spectra/second for MS/MS. During LC-MS analysis, reference masses (m/z 121.050873 and m/z 922.009698) were monitored continuously for accurate mass calibration. Targeted MS/MS analysis was conducted for SAR (precursor ion m/z 542.2) and the internal standard (precursor ion m/z 392.2), both using a narrow isolation window and a collision energy of 25 eV. Data processing and quantification were performed using Agilent MassHunter Qualitative Analysis and Agilent MassHunter Quantitative Analysis software (version 10.0). SAR was quantified based on extracted ion chromatograms at transitions m/z 542.2→127.1218 ± 50 ppm for SAR, and m/z 392.2→246.1143 ± 50 ppm for the internal standard. Quantification involved normalization to the internal standard and calibration against a linear SAR standard curve, adjusted for sample mass and volume. The limits of detection (LOD) and quantification (LLOQ) were established using the signal-to-noise ratio and confirmed through spiked calibration standards.

### Nitrite assay

Nitrite concentrations were quantified using the Griess reaction assay. Briefly, 50 μL of serum sample was added to each well of a 96-well plate, followed by an equal volume (50 μL) of Griess reagent (Sigma; catalog # 03553). The mixture was incubated at room temperature for 10 minutes. Following incubation, absorbance was measured at 540 nm using a Synergy 2 multi-mode microplate reader (BioTek Instruments, USA). Nitrite concentrations in the samples were determined from a standard calibration curve generated using known nitrite concentrations. Results are expressed as mean ± SEM.

### Reactive oxygen species (ROS) assay

Reactive oxygen species (ROS) levels in serum samples were measured using a dichlorodihydrofluorescein (DCF) ROS/RNS Assay Kit (Abcam; catalog # ab238535), following the manufacturer’s instructions. Fluorescence intensity was quantified at an excitation wavelength of 480 nm and an emission wavelength of 530 nm using the Synergy 2 multi-mode microplate reader (BioTek Instruments, USA). ROS concentrations were calculated using a hydrogen peroxide (H_2_O_2_) standard curve, and results are reported in relative fluorescence units (RFUs).

### Glutathione assay

Glutathione (GSH) and oxidized glutathione (GSSG) levels were quantified using the Glutathione assay kit (Invitrogen; catalog # EIAGSHC) following the manufacturer’s guidelines. Briefly, serum samples were prepared in two conditions: one set was treated with 2-vinylpyridine (2-VP) to selectively measure oxidized glutathione (GSSG) by blocking reduced glutathione (GSH) from contributing to the colorimetric reaction. Another set of samples was not treated with 2-VP, allowing the measurement of total glutathione (GSH + GSSG). Following reagent addition, samples were incubated in a 96-well plate according to kit instructions, and absorbance was recorded at 405 nm using a Synergy 2 multi-mode microplate reader (BioTek Instruments, USA). Concentrations of GSH and GSSG were calculated against a standard curve. The concentrations of free GSH, oxidized GSSG, and the ratio of GSH to GSSG were analyzed and expressed in μM.

### Immunohistochemical analysis, imaging, and quantification of brain tissue

Animals were euthanized at 18 weeks following soman exposure. Under terminal anesthesia, animals were perfused transcardially with 4% paraformaldehyde (PFA). Blood samples were collected prior to perfusion. Extracted brains were fixed in 4% PFA for 24 hours, followed by cryoprotection in 25% sucrose solution for 72 hours at 4°C. The brains were then embedded in gelatin, rapidly frozen in liquid nitrogen cooled, and stored at −80°C until sectioning. Coronal sections (16 μm thick) were cut using a cryostat (ThermoFisher) and mounted onto glass slides. Each slide contained five sections spaced approximately 480 μm apart, representing rostral to caudal brain regions. Unstained sections were stored at − 20°C, while stained sections were kept at 4°C. For immunohistochemistry (IHC), antigen retrieval was performed using citrate buffer at 95°C for 23 minutes. Sections were then transferred into Shandon racks, washed five times with PBS, and blocked in 10% normal donkey serum for one hour at room temperature. Sections were incubated overnight at 4°C with primary antibodies. After primary antibody incubation, sections were washed five times in PBS, incubated with fluorochrome-conjugated secondary antibodies for one hour at room temperature, and again washed five times in PBS. Details of antibodies are provided in Table 4. Slides were mounted using VectaShield mounting medium containing DAPI. Imaging was performed using a Leica DMi8 inverted fluorescence microscope equipped with a Leica K5 passive-cooled sCMOS camera. Five sections per animal were imaged, covering distinct brain regions. Immunopositive cells were counted from each brain region per section, and data were averaged and graphed separately for each region.

### NeuN and Fluoro-Jade B (FJB) double staining for neurodegeneration analysis

To assess neurodegeneration, brain sections previously immunostained for NeuN were further processed using Fluoro-Jade B (FJB) staining. Sections were first incubated in 0.006% potassium permanganate solution for 5 minutes with gentle agitation, followed by three brief rinses (1 minute each) in distilled water. Subsequently, slides were incubated in 0.0003% FJB solution prepared in 0.1% acetic acid for 10 minutes in the dark, followed by three additional rinses in distilled water (1 minute each). Slides were then air-dried in the dark at room temperature, cleared in xylene, and mounted using Surgipath Acrytol (Leica Biosystems, IL). Co-labeled neurons exhibiting NeuN (red) and FJB (green) fluorescence were manually counted across five sections per animal. Mean cell counts were calculated, plotted, and analyzed statistically.

### Glial scar volumetric quantification by Cavalieri (Trapezoidal) Approximation

Volumes of glial scars were quantified using image analysis software from serial brain tissue sections. Scar areas (μm^2^) were measured individually for each tissue section. The Cavalieri (trapezoidal) approximation method was applied to estimate the scar volume between consecutive sections. This method involved calculating the volume between each pair of consecutive sections using the formula: V = [(A1 + A2)/2] × d, where A1 and A2 represent scar areas of two consecutive sections, and d is the known distance (480 μm) between these sections. Volumes calculated from all consecutive pairs of sections were summed to determine the total scar volume per animal. This approach assumed linear interpolation between sections and provides a reliable estimation of total lesion volume. Statistical analyses of the resulting scar volumes were performed to compare treatment effects (Stehr et al. 2022).

### Statistical analysis

Statistical analyses and experimental design guidance were provided by Dr. Wang, a biostatistician at the College of Veterinary Medicine, Iowa State University. All samples were randomized, and experimental groups were blinded until data analysis completion. GraphPad Prism 10.0 software was employed for statistical analyses. Robust Estimation of Outlier (ROUT) tests were conducted where appropriate. Normality was assessed using the Shapiro Wilk test. Depending on data normality, comparisons between two groups were performed using either unpaired t-tests or Mann Whitney tests. Interactions between sex and treatment were evaluated using two-way ANOVA with appropriate post-hoc tests for multiple comparisons. If significant interaction effects were detected, data were presented separately by sex with relevant post-hoc analyses. In the absence of significant interactions, data from both sexes were pooled. Pearson correlation coefficients were used to compute correlations, while simple linear regression was applied for slope comparisons. Data in this manuscript was either presented as Mean ± SEM except Cohen’s d effect size in Fig. 9e (Ho et al. 2019).

### Rigor, sample size, and inclusion or exclusion criteria

Animals were randomized irrespective of sex, or body weight into different treatment groups. Age and sex-matched controls, as well as severity-matched soman-exposed groups, were blinded. Sample size and statistical power were determined based on prior OPNA study outcomes related to gliosis and neurodegeneration at a 95% confidence interval. Animals that failed to achieve the set SE severity score threshold (> 20 minutes of SE) or did not survive soman exposure (132 μg/kg, s.c., approximately 1.2 × LD50) were excluded. Animals suspected of dosing errors were also excluded. Animals exhibiting significant weight loss (> 20% within 3–4 days post-exposure) or signs of severe distress (e.g., pain behaviors, lack of grooming, severe motor impairments) were euthanized and excluded from the study. Based on these criteria, three female rats that died post-exposure were excluded from analysis.

## Results

### Soman exposure induced severe SE in male and female rats, and the consumption of SAR-in-diet reached the targeted dose that strongly correlated with serum SAR concentrations.

**Figure 2:**
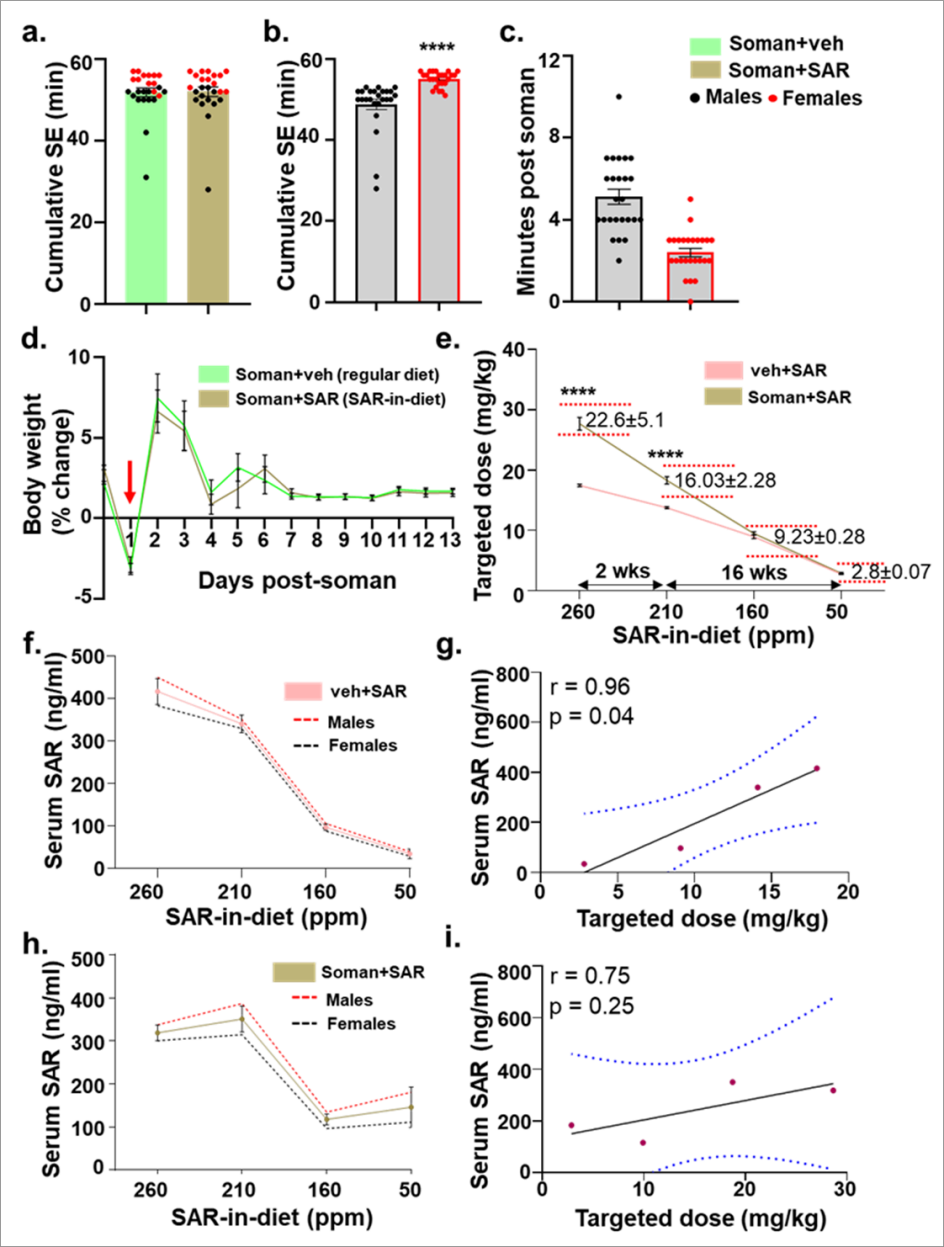
Development of SE and body weight recovery after acute soman exposure, with a correlational analysis between SAR-in-diet and serum SAR concentrations. Following acute soman exposure, rats were observed, and the initial SE severity was quantified. SE severity (*minutes of convulsive seizures between soman exposure and midazolam treatment*) is shown in both treatment groups as a mixed sex cohort (a) or as separate sexes (b). The SE severity grouping and comparison (in panel ‘a’) was done before the animals were coded/blinded and treated with SAR or vehicle. (c) Latency to the first convulsive seizure in all males and females after soman exposure is shown. (d) Body weight gain progression in rats following soman exposure (day 0) fed with diet with or without SAR is shown. (e) A comparative analysis of targeted SAR dose (mg/kg) achieved was based on SAR-in-diet consumption shown in different groups as mixed-sex cohorts. (f-i) Serum SAR concentrations (ng/ml), estimated by LC/MS, compared with SAR-in-diet concentrations (ppm) in vehicle (f) and soman-exposed (h) animals, and their correlation analyses (g, i). Normality of the data was assessed with the Shapiro-Wilk test. Bars represent mean ± SEM, and each dot on the bar graphs represents a data point for each animal. (f-i) n = 25 (12–13/sex) (a-e) & n = 9–10 (4–5/sex). (b) ****p < 0.0001 by Unpaired t-test, (e) ****p<0.0001 by two-way ANOVA with Tukey’s multiple comparisons & (g) *p<0.05, by Pearson correlation analysis. No sex differences were observed (except b and c), and the data from both sexes was pooled.

Following acute exposure to soman, all animals developed severe SE (> 25 minutes), irrespective of sex and the stages of the estrous cycle, and were randomly distributed to treatment groups (Fig. 2a). Interestingly, females had a significantly higher SE severity score compared to males (Fig. 2b). The latency to the onset of first CS following acute exposure to soman was also significantly lower in female rats (Fig. 2c). A transient decline in body weight was observed in all animals one day post soman exposure (indicated by a downward red arrow), which was followed by a quick recovery (Fig. 2d). Animals on SAR treatment (soman + SAR and vehicle + SAR) received SAR orally in the first week followed by SAR-in-diet for 18 weeks in a tapering dosing regimen. During the 18 weeks, animals exposed to soman consumed more SAR-incorporated diet and gained more weight when compared to non-exposed animals (Supplementary Fig. 1). Additionally, the targeted SAR dose achieved in the control group (vehicle + SAR) was lower during the first 2 weeks of feeding SAR-in-diet but achieved comparable targetted dose range over a chronic feeding timeline from weeks 3–18 (Fig. 2e). No significant sex differences were observed in targetted doses within each treatment group (Supplementary Fig. 2). Additionally, the serum concentrations of SAR showed a strong correlation with the corresponding SAR-in-diet concentration in both treatment groups (Fig. 2f–i).

### SAR mitigated soman-induced anxiety-like behavior at 5–6 weeks post-exposure

**Figure 3:**
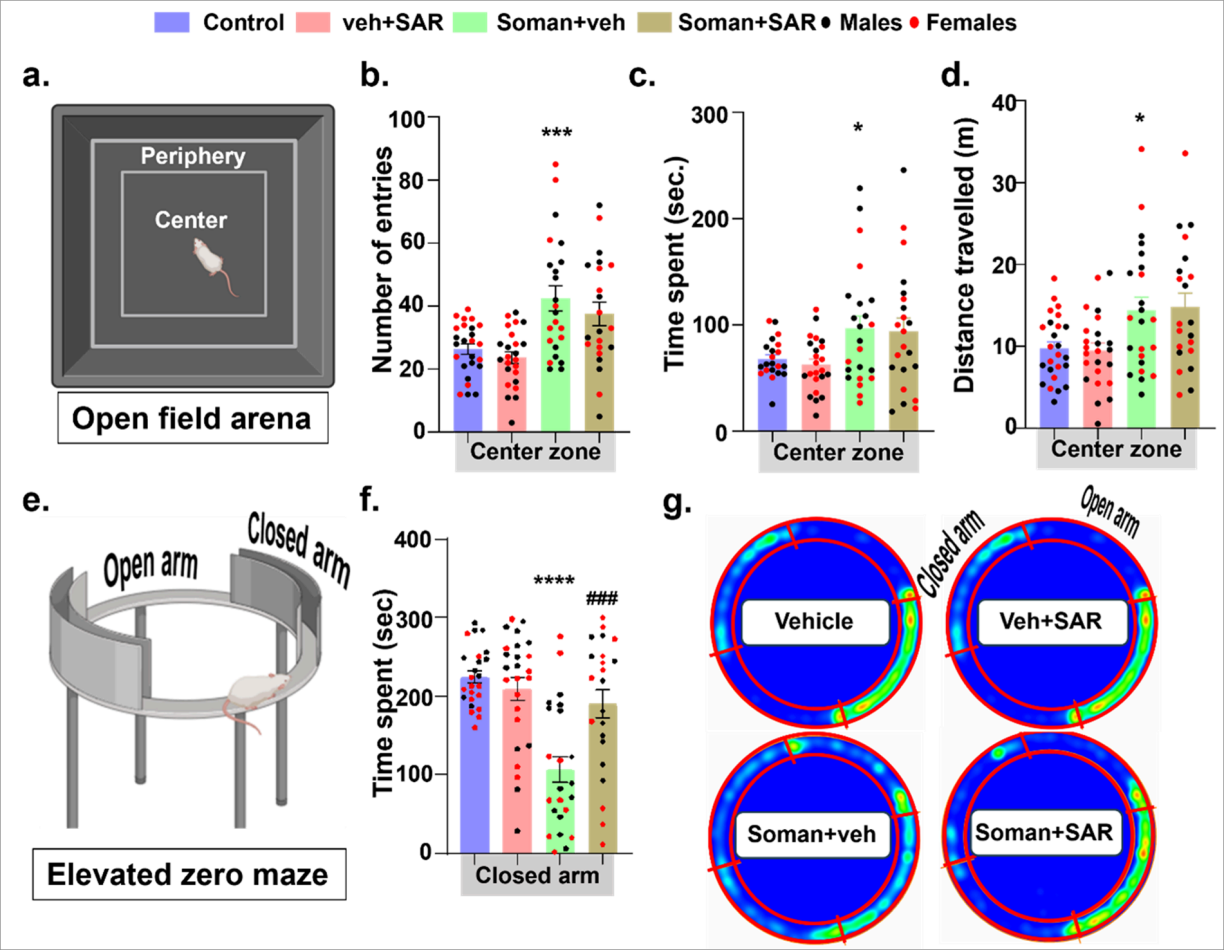
Anxiety and exploratory behaviors (risk assessment) in rats at 5–6 weeks following soman exposure. Results from the (a) open field test and (e) elevated zero maze at 5–6 weeks post-soman are presented. (b-d) The results from the open field test plotted as the number of entries, the time spent, and the distance traveled in the central zone. (f) The quantified results from the elevated zero maze are plotted as the time spent in the closed arm. (g) Representative heatmaps of the time spent at each zone are shown. Normality was assessed with the Shapiro-Wilk test. *indicates the soman effect (soman vs control) and # represents SAR effect (soman+SAR vs soman+vehicle). Bars represent mean ± SEM and each dot on the bar graphs represent a data point for each animal. n = 22–25 (10–13/sex). *p<0.05, ***p<0.001, ****p<0.0001, and ^####^p<0.0001 by two-way ANOVA with Tukey’s multiple comparisons. No sex differences were observed, therefore data from males and females were pooled.

An open field arena divided into the peripheral and center zones was utilized to test the anxiety-like behavior, and it also served as a broad measure of activity and exploratory drive (Fig. 3a). Animals exposed to soman had significantly a greater number of entries, spent more time, and travelled more distance in the center zone compared to control group. SAR treatment had no effect on soman-induced behavioral changes (Fig. 3a–d). We also employed the elevated zero maze to evaluate compromised anxiety-like behavior and their risk-assessment behavior, based on the premise that exposure to an elevated open platform generally elicits heightened anxiety responses (Fig. 3e). Soman exposed animals without SAR treatment deviated from their natural behavior (avoiding risk) and spent more time in the open arms compared to the control group, while SAR-in-diet treatment recovered the natural behavior in soman-exposed rats, which spent significantly more time in the closed arms than the open arms in contrast to soman-vehicle group (Fig. 3f). A cumulative exploratory behavior shown in heatmaps for each group illustrates soman-induced effect and mitigation by SAR treatment (Fig. 3g).

### SAR did not rescue the soman-induced deficit in recognition memory at 5–6 weeks post exposure.

**Figure 4:**
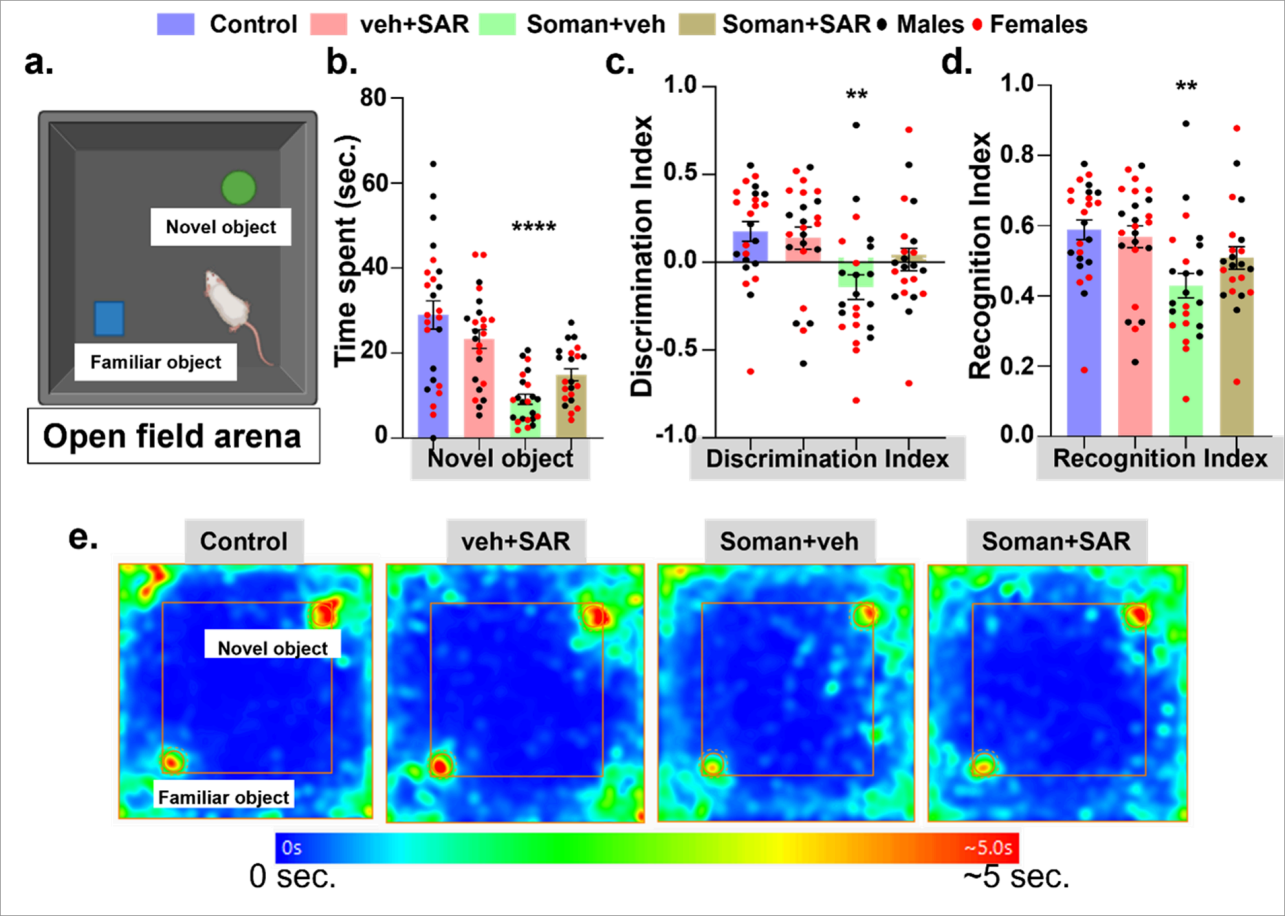
Recognition memory profile in rats following soman exposure. (a) Open field arena with objects of different shapes and colors were used to test the recognition memory in rats at 5–6 weeks post soman. (b-d) The results are plotted as the time spent with the novel object, discrimination index, and recognition index. (e) Representative heatmaps of time spent in the arena. Normality was assessed with the Shapiro-Wilk test. The stars indicate soman effect (soman vs control). Bars represent mean ± SEM and each dot on the bar graphs represent a data point for each animal. n = 22–25 (10–13/sex). **p<0.01, and ****p<0.0001 by two-way ANOVA with Tukey’s multiple comparisons. No sex differences were observed, therefore the data from males and females were pooled.

Recognition memory was analyzed using a novel object recognition test over 2 days in an open field arena where animals were given a two familiar objects on day 1 and a novel object along with one familiar object on day 2 (Fig. 4a). Animals exposed to soman spent significantly less time with the novel object on day 2 (Fig. 4b). Soman-exposed animals also had low scores on discrimination and recognition indices while SAR treatment did not improve these indices significantly (Fig. 4c–d). A higher index typically reflects better recognition memory and a stronger preference for novelty. A cumulative heatmap representing each group shows the exploratory behavior of rats with the two objects, where soman exposed animals, treated regardless of SAR or vehicle, had less interaction with the novel object compared to the control group (Fig. 4e).

### SAR restored motor deficits but did not mitigate deficit in contextual/cued memory at 6–7 weeks post soman exposure.

**Figure 5:**
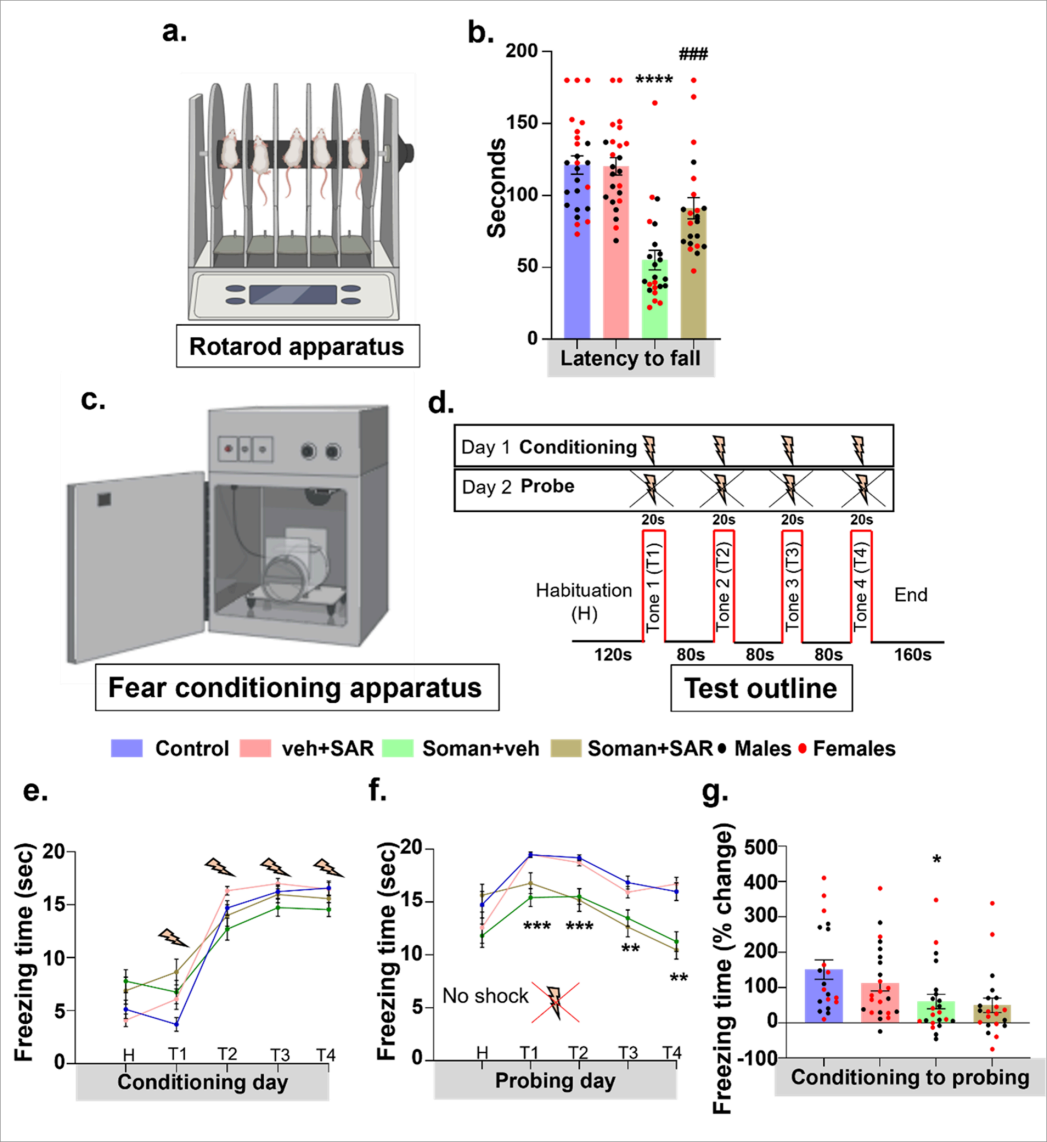
Motor behavior and contextual memory profiles after soman exposure. (a) Rotarod test was used to analyze the motor behavior in rats at 6–7 weeks post soman exposure. (b) Results from different treatment groups are plotted as latency to fall, which is the time taken by the rat to fall from an accelerating rotating rod, (c) Fear conditioning test was carried out to examine contextual memory in rats. The experimental paradigm for the test is illustrated in panel ‘d’. On day 1, animals were conditioned with four consecutive 20s tones (T1-T4) each with a 80 second interval between trials. (f) On the test day 2, after habituation, all four tones were repeated with similar intervals but without electric shock. (e-g) The data from the conditioning and testing/probe sessions are plotted as freezing duration (in seconds) for each animal during the 20-second tone. The results were analyzed as the percent change in freezing time from day 1 to day 2 for each tone, and the average percent change was calculated for each animal. Normality was assessed with the Shapiro-Wilk test. The stars indicate soman effect (soman vs control) and # represents SAR effect (soman+SAR vs. Soman+vehicle). Bars represent mean ± SEM and each dot on the bar graphs represent a data point for each animal. (b) n = 22–25 (10–13/sex). ****p<0.0001, and ^####^p<0.0001 by two-way ANOVA with Tukey’s multiple comparisons. (f-g) *p<0.05, **p<0.01, and ***p<0.001 by two-way ANOVA with Tukey’s multiple comparisons. No sex differences were observed, therefore the data from males and females were pooled.

Rotarod was used to test motor skills where rats from each group were placed on a rotating rod for habituation/training on day 1 and day 2 (Fig. 5a). The actual testing for each animal was conducted on day 3 using an accelerated protocol, consisting of three trials, each lasting three minutes. The trial concluded either at the end of three minutes or when an animal falls during the three minutes run. No second chance was given if an animal falls. Soman-exposed rats also exhibited hyperesthesia, reacted intensely to touch, sound, and sudden movements, which impaired their ability to stay on the rotarod. Compared to controls, soman-exposed animals on control diet (without SAR-in-diet treatment) demonstrated significantly shorter durations on the accelerating rotarod. In contrast, soman exposed animals that were fed on SAR-in-diet significantly performed better by spending longer time on the rotating rod and showed higher latency to fall (Fig. 5b). The fear conditioning apparatus was used to test the contextual memory (Fig. 5c), where animals were conditioned with a tone cued with a shock on day 1 and tested for the cued memory on day 2 when only tone was presented without a shock (Fig. 5d). On day 1, the freezing times at tone 2, 3 and 4 were higher, when compared to the habituation and tone 1 (as there was no prior experience with a shock paired to a tone) (Fig. 5e). On the day of testing/probing (day 2), soman exposed animals with or without SAR-in-diet showed lower freezing time when compared to non-exposed control animals, indicating a deficit in contextual/cued memory due to soman exposure and SAR treatment had no effect (Fig. 5f–g).

### SAR treatment showed short-term benefits by restoring hippocampal connectivity at 8–9 weeks and more widespread protective effects by 18 weeks post-soman.

**Figure 6:**
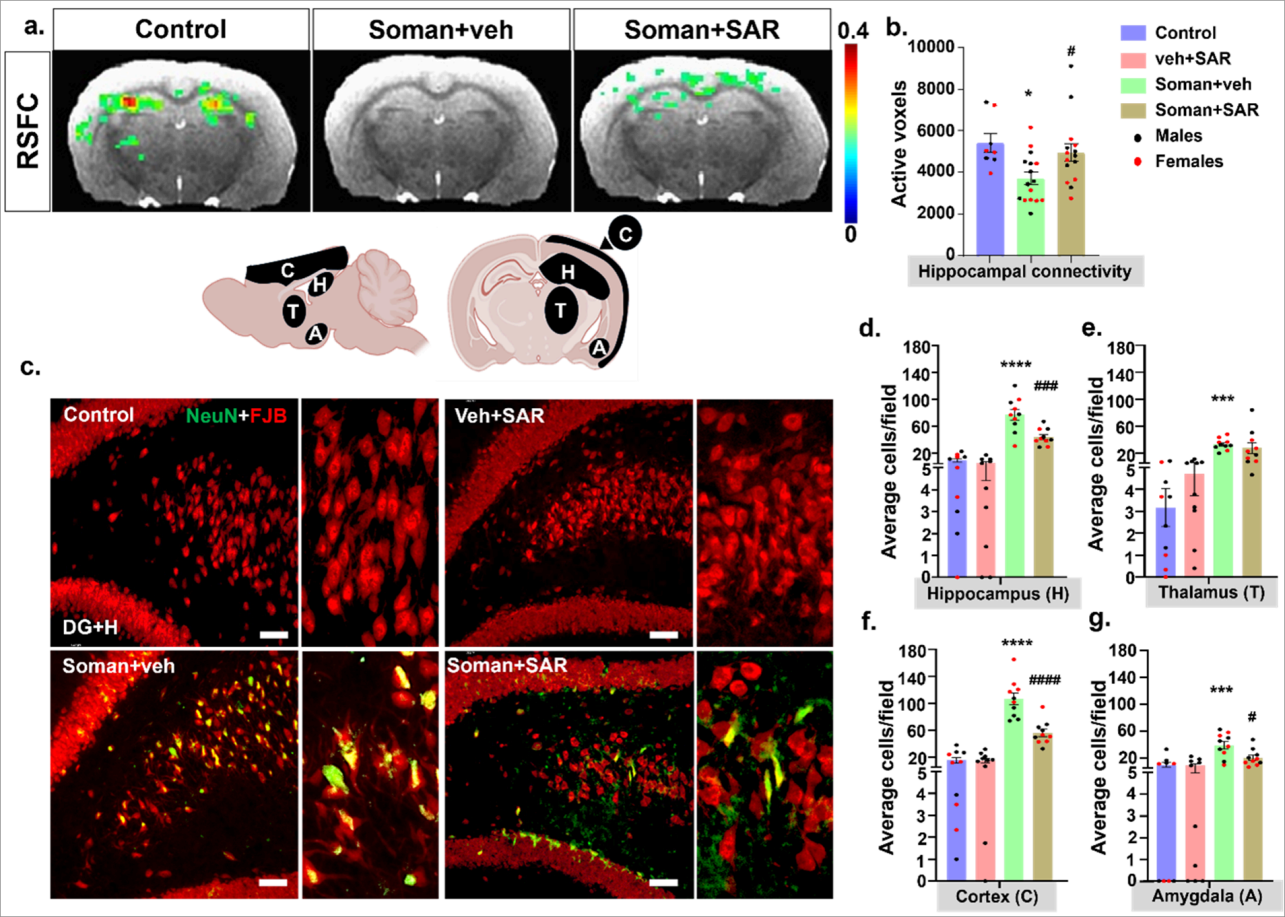
SAR mitigated soman-induced hippocampal functional connectivity, measured by resting state fMRI, and neurodegeneration. (a) Resting state functional connectivity (RSFC) of the hippocampal network, measured by fMRI, are presented as color overlay activation maps across each experimental group along with the anatomical MRI underlay (the scale bar represents the correlation strength of the active voxels with the hippocampal seed region). (b) The number of active voxels in the hippocampal functional connectivity map derived from each animal are displayed in different treatment groups. (c) Representative immunohistochemistry (IHC) images from the hippocampus with FJB-positive cells (green) and NeuN (red, neuronal marker) are shown. (d-g) Quantification of FJB & NeuN positive cells from the hippocampus, thalamus, cortex, and amygdala. *indicates soman effect (soman vs control) and # represents SAR effect (soman+SAR vs Soman+vehicle). Normality was assessed with the Shapiro-Wilk test. Bars represent mean ± SEM and each dot on the bar graphs represent a data point for each animal. (a) Color of the voxels represent the correlation strength of that voxel with the location. (a-b) n = 8–16 (4–8/sex) *p < 0.05 by two-way ANOVA with Tukey’s multiple comparisons. (c-e) n = 10 (5/sex). */^#^p < 0.05, **/^##^p < 0.01, ***/^###^p < 0.001, ****/^####^p < 0.0001 by two way ANOVA (mixed effects model) with Tukey’s multiple comparisons, scalebar=100 μm. No sex differences were observed, therefore data were pooled. Individual analysis from Dentate gyrus + Hilus (DG+H), CA1, CA3, Subiculum (SUB), medial dorsal thalamus (MDT), motor cortex (MC), Somatosensory cortex (SSC), and Piriform Cortex (PC) can be found in Supplementary figure 3.

Functional MRI scans at 8–9 weeks post-soman exposure revealed significant loss of functional connectivity in the hippocampus in the vehicle group. SAR treatment significantly protected hippocampal functional connectivity (Fig. 6a, 6b). Immunohistochemistry of the brain sections co-labeled with FJB and NeuN to capture degenerating neurons (Fig. 6c) at 18 weeks post-soman confirmed a significant increase in neurodegeneration in the hippocampus, thalamus, cortex, and amygdala of the soman-exposed vehicle-treated group. SAR treatment significantly mitigated soman-induced neurodegeneration in all regions except the thalamus (Fig. 6d–g).

### SAR long-term treatment modulated the soman-induced severity of convulsive and non-convulsive seizures, their duration, and the epileptiform discharges

**Figure 7:**
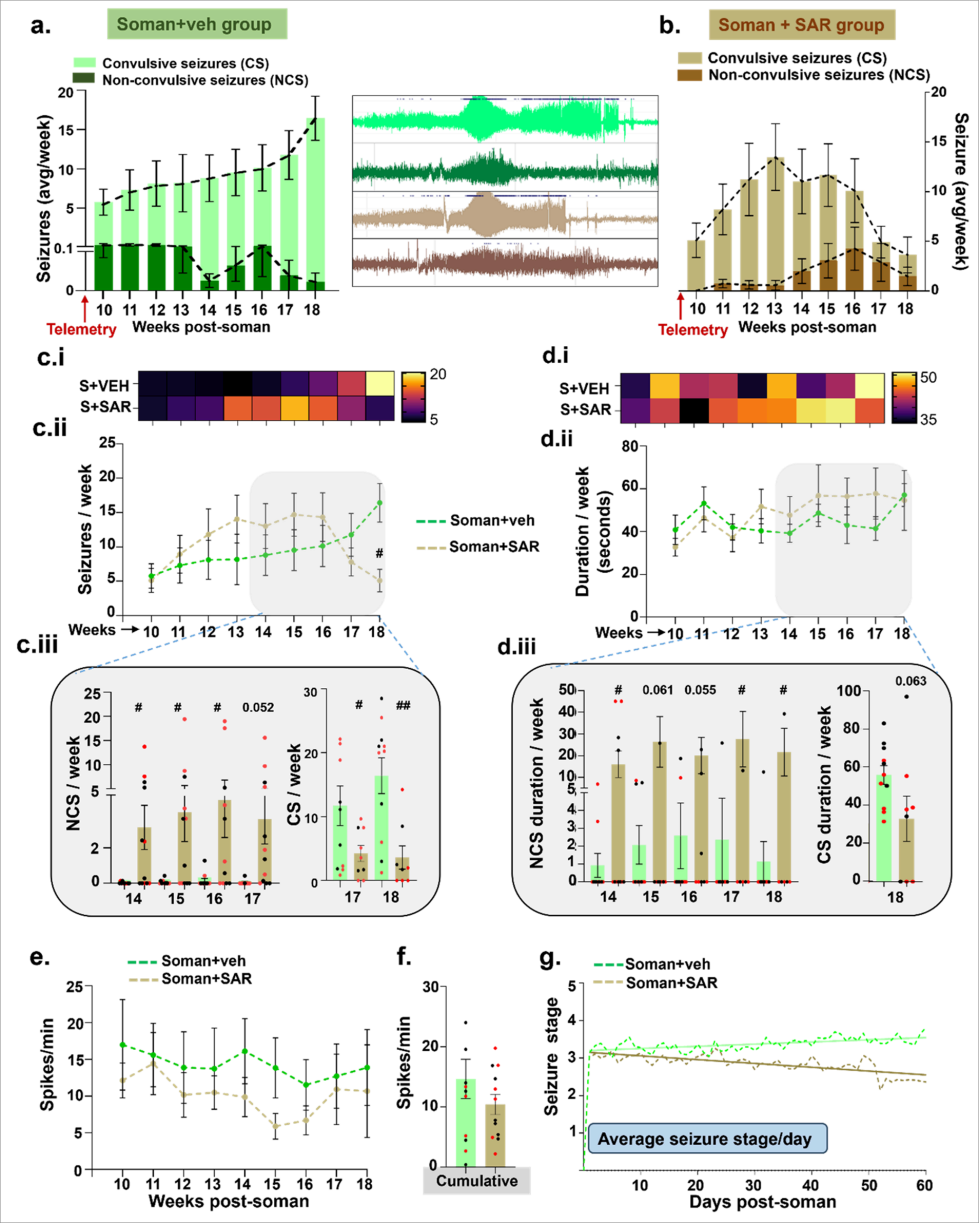
Seizure characteristics in rats following soman exposure in vehicle and SAR treated groups. Rats exposed to soman were implanted with telemetry devices for continuous video EEG recording. (a-b) Trends of convulsive seizures (CS) and non-convulsive seizures (NCS) within each treatment group with their representative EEG traces are shown. A comparative analysis of the total number of seizures is presented as heatmaps (c.i, d.i) to illustrate relative differences between groups, and (c.ii, d.ii) line graphs showing weekly progression over time. (c.iii, d.iii) Breakdown of total seizure numbers into non-convulsive and convulsive seizures. Number of epileptiform spikes is shown as weekly progression (e) and cumulative bar graph (f) for all weeks. (g) The daily average seizure stage representing seizure severity for each group is presented. Normality was assessed with the Shapiro-Wilk test. # represents SAR effect (soman+SAR vs Soman). Bars represent mean ± SEM and each dot on the bar graphs represent a data point for each animal. n = 8–12 (3–6/sex), ^#^p < 0.05, ^##^p < 0.01 by two-way ANOVA (mixed effects model) with Sidak’s multiple comparisons. Color in the heatmap represents the number (c) and duration of average seizures (d) per week. No sex differences were observed, therefore the data were pooled.

Following continuous video-EEG acquisition for 60 days from week 10 to 18 weeks post-soman, we examined each type of spontaneously recurring convulsive and non-convulsive seizures based on the integrated videos and associated changes on the EEG. After a rigorous analysis of all the seizure events across all groups, we categorized each seizure based on severity (convulsive and non-convulsive) and duration. We also quantified the epileptiform spikes. A few examples are shown in Fig. 7a–b. Although the total number of seizures did not differ significantly between soman-exposed animals with or without SAR treatment (except at week 18), a shift in seizure severity was observed in the SAR-treated group. Specifically, after 14 weeks post-soman exposure, SAR-treated animals showed a significantly higher number of non-convulsive seizures and fewer convulsive seizures (Fig. 7c). A similar trend was observed in seizure duration after 14 weeks post-soman exposure. In SAR-treated animals, the duration of non-convulsive seizures increased proportionally, while the duration of convulsive seizures decreased. However, the combined average duration of all seizures (non-convulsive and convulsive) did not change between groups (Fig. 7d). No significant reduction in the epileptiform spikes was observed in soman exposed animals treated with or without SAR-in-diet during the 10–18 weeks (7e-f). Overall, long-term SAR treatment reduced seizure severity following soman exposure, as reflected by a decrease in the average seizure stage over time (Fig. 7g).

### SAR treatment protected the soman-induced loss of inhibitory neurons

**Figure 8:**
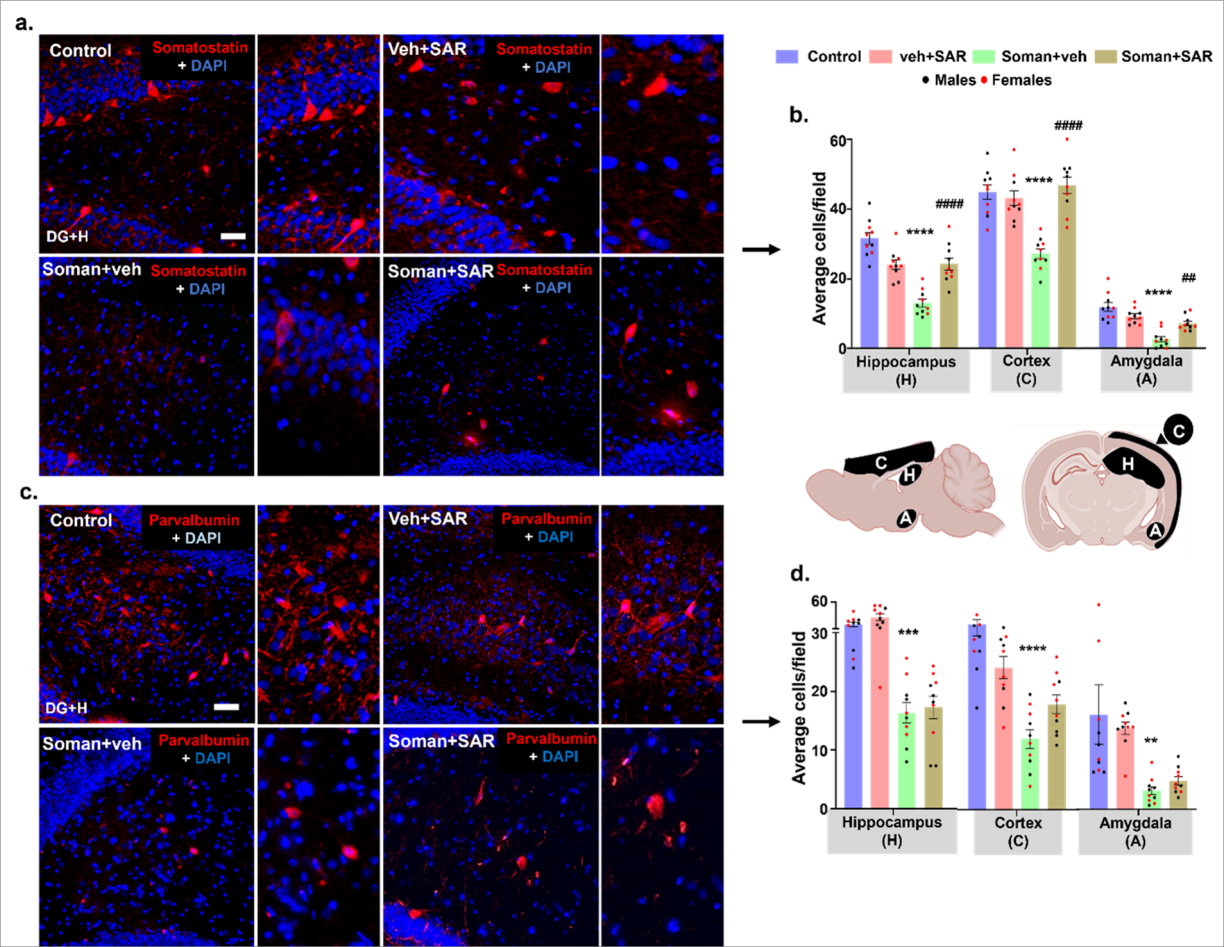
SAR effect on soman-induced loss of parvalbumin and somatostatin inhibitory neurons. Representative images of (a) parvalbumin (red) and (b) somatostatin (red) with DAPI (blue, nuclear stain) from the dentate gyrus (DG)+ hilus, a part of the hippocampus, are shown. Cell quantification- for parvalbumin and (d) somatostatin-positive neurons from the hippocampus, cortex, and amygdala are shown. Normality was assessed with the Shapiro-Wilk test. *indicates soman effect (soman vs control) and # represents SAR effect (soman+SAR vs Soman). Bars represent mean ± SEM and each dot on the bar graphs represent a data point for each animal. n = 10 (5/sex). */^#^p < 0.05, **/^##^p < 0.01, ***/^###^p < 0.001, ****/^####^p < 0.0001 by two- way ANOVA (mixed effects model) with Tukey’s multiple comparisons (B-E), scalebar=100 μm. No sex differences were observed, therefore the data were pooled. Individual analysis from Dentate gyrus+Hilus (DG+H), CA1, CA3, Subiculum (SUB), motor cortex (MC), Somatosensory cortex (SSC), and Piriform Cortex (PC) can be found in Supplementary figure 4.

We further investigated the impact of soman exposure on somatostatin and parvalbumin inhibitory neurons that are most vulnerable to initial seizures. Representative images from somatostatin (Fig. 8a) and parvalbumin (Fig. 8c) positive neurons from the hippocampus are presented from all treatment groups. A loss of somatostatin and parvalbumin was observed in the hippocampus, cortex, and amygdala of the soman-exposed animals (Fig. 8b&d). SAR treatment significantly protected somatostatin inhibitory neurons in all three regions (Fig. 8b). However, parvalbumin inhibitory neurons were partially protected in the motor cortex but not in the hippocampus and amygdala (Fig. 8d).

### SAR treatment reduced soman-induced glial scars

**Figure 9:**
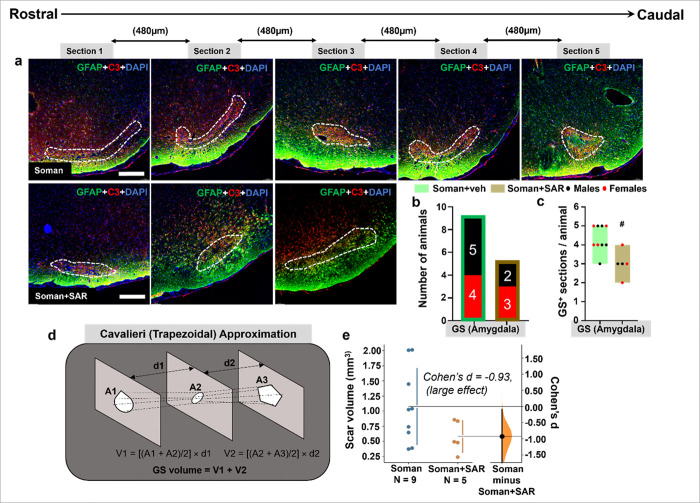
Gilal scar quantification in the amygdala of soman exposed rats treated with or without SAR. (a) Representative images of Glial scars from the amygdala. tThe brain sections were co-labeled for GFAP and C3 and counterstained with DAPI (blue, nuclear stain). (b-c) The total number of rats (males and females) positive for glial scars and the number of glial scar-positive sections for each animal are shown in the bar graph. (d-e) Cavalieri (Trapezoidal) Approximation was used for volumetric analysis on sequential sections, and Cohen’s d estimation for effect size estimation between treatment groups. Normality was assessed with the Shapiro-Wilk test. # represents SAR effect (soman+SAR vs Soman). Each dot on the bar graphs represents a data point for each animal.(c-d). n=9 (5 males/4 females) for soman+veh and n=5 (2 males/3 females) for soman+SAR. ^#^p < 0.05 by Mann Whitney U test (c), effect size by Cohen’s d analysis (e), Scalebar=100 μm. No sex differences were observed, therefore data were pooled.

Five 16μm serial brain sections, each separated by 480μm, were sampled for glial scar quantification. Co-labeling with GFAP + C3 revealed glial scars (filled with degenerating neurons and reactive microglia) in the amygdala that were surrounded by reactive astrocytes in the periphery of the scars in soman exposed vehicle group. The representative images of the glial scar from the amygdala are shown in Fig. 9a. A total of 9 rats (5 male and 4 female) in soman + vehicle group and 5 rats (2 males and 3 females) in the soman + SAR group had glial scars in the amygdala (Fig. 9b). Analysis of glial scar positive sections in each animal revealed a significant difference between the soman vehicle aoman and SAR-treated groups (Fig. 9c). The area of glial scar in each section was measured to calculate the total glial scar volume using Cavalieri (Trapezoidal) Approximation (Fig. 9d). Cohen’s d estimation of glial scar volume indicated a large negative effect size in SAR-treated animals indicating a reduction in the glial scar volume (Fig. 9e).

### SAR treatment significantly reduced soman-induced reactive gliosis

**Figure 10:**
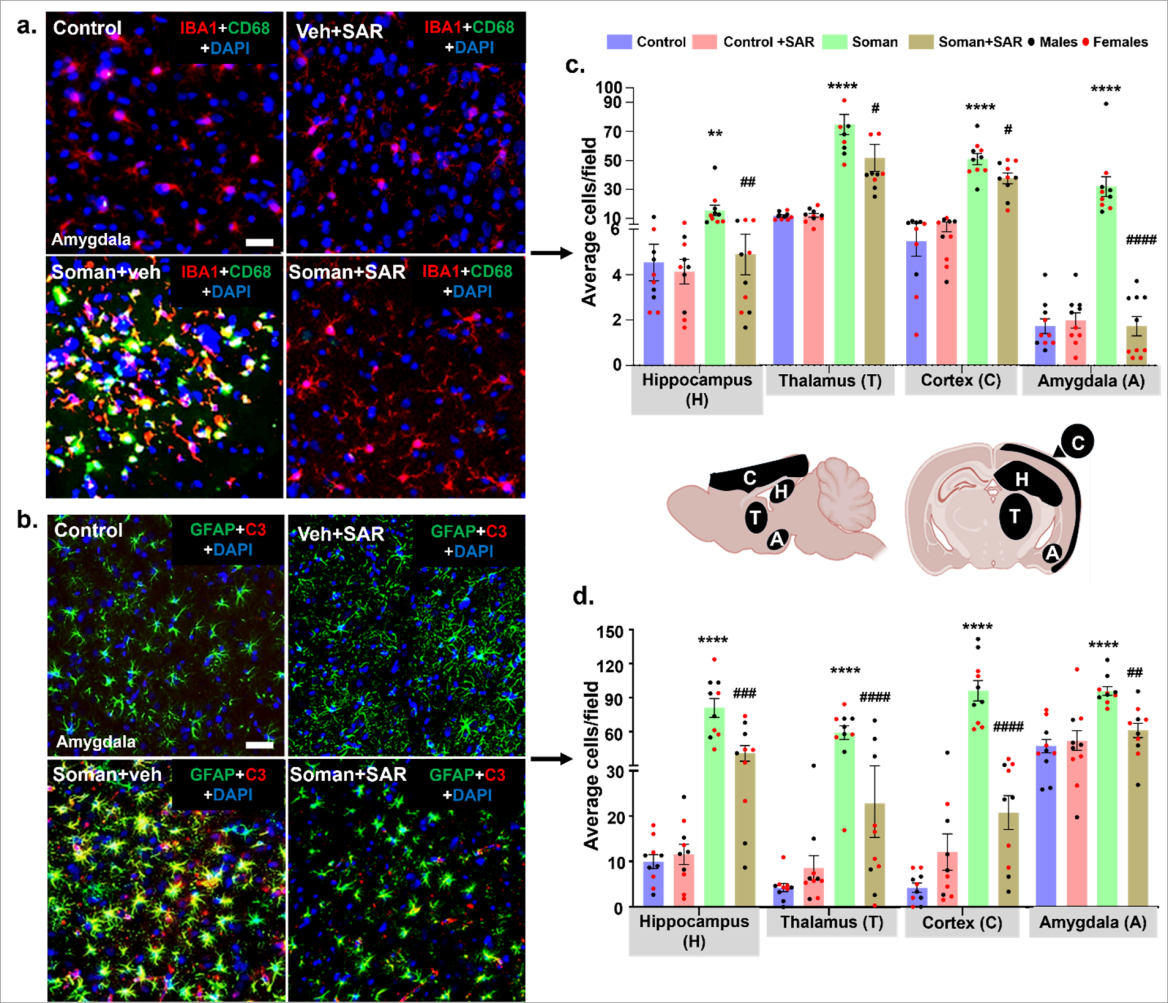
Reactive gliosis following soman exposure and mitigation by SAR. (a) Reactive microgliosis, representative images of IBA1 (red) and CD68 (green) positive cells and DAPI (blue, nuclear stain) immunostaining from the amygdala are shown. (c) Cell quantification for IBA1+CD68 co-labeled cells from the hippocampus, thalamus, cortex, and amygdala are shown. (b) Reactive astrogliosis-representative images of GFAP (green) and C3 (red) positive cells and DAPI (blue, nuclear stain) immunostaining from the amygdala are shown. (d) Cell quantification for GFAP+C3 co-labeled cells from the hippocampus, thalamus, cortex, and amygdala is shown. Normality was assessed with the Shapiro-Wilk test. *indicates soman effect (soman vs control) and # represents SAR effect (soman+SAR vs Soman). Bars represent mean ± SEM, and each dot on the bar graphs represents a data point for each animal. (c-d) n = 10 (5/sex). */^#^p < 0.05, **/^##^p < 0.01, ***/^###^p < 0.001, ****/^####^p < 0.0001 by two way ANOVA (mixed effects model) with Tukey’s multiple comparisons. No sex differences were observed. Scalebar=100 μm. Individual analysis from the dentate gyrus + hilus (DG+H), CA1, CA3, Subiculum (SUB), lateral dorsal thalamus (LDT), medial dorsal thalamus (MDT), Ventral posteromedial thalamus (VPM) motor cortex (MC), Somatosensory cortex (SSC), and Piriform Cortex (PC), can be found in Supplementary figure 5.

with the brain sections were immuno-stained for IBA1 + CD68 to identify microglia (IBA1) with reactive phenotype (CD68, a phagocytic marker) and GFAP + C3 to target astrocytes (GFAP) with reactive phenotype (C3, a marker of inflammation). Representative images from the amygdala are shown from all treatment groups (Fig. 10a–b). Soman exposure induced a significant increase in the reactive microglia and reactive astrocytes in the hippocampus, thalamus, cortex, and amygdala compared to control. SAR-treated soman-exposed rats displayed a significantly lower number of reactive microglia and reactive astrocytes in all regions (Fig. 10c–d).

### SAR mitigated soman-induced nitro-oxidative stress at 18 weeks post- exposure

**Figure 11:**
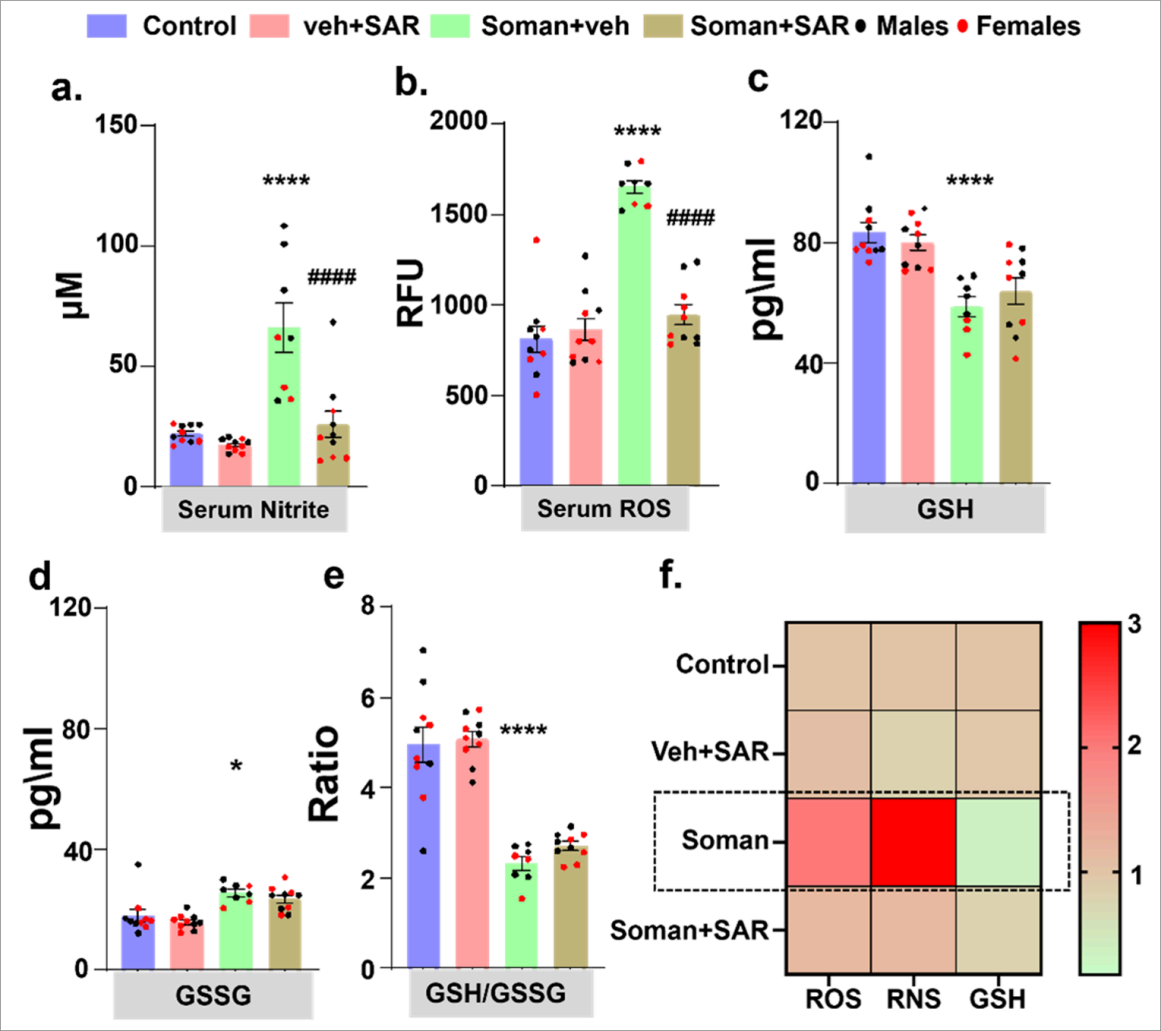
Nitro-oxidative stress markers, and glutathione antioxidant levels in the serum at 18 weeks post-exposure and the effects of SAR. Griess nitrite assay kit, ROS assay kit, and Glutathione assay kit were used to determine the concentrations of nitrite, ROS, and GSH/GSSG levels in the serum. The results are plotted as (a) μM for serum nitrite, (b) relative fluorescence units (RFU) for ROS, and (c-e) as pg/mL or as a ratio for serum glutathione (GSH/GSSG). (f) Fold changes in these markers, compared to the control (at 1), are displayed as a heatmap. Normality was assessed with the Shapiro-Wilk test. Stars indicate soman effect (soman vs control) and # represents SAR effect (soman+SAR vs Soman+vehicle). Bars represent mean ± SEM and each dot on the bar graphs represent a data point for each animal. n = 10 (5/sex). ****/^####^p < 0.0001 by two-way ANOVA with Tukey’s multiple comparisons. No sex differences were observed, therefore the data from both sexes were pooled.

Serum analysis of nitro-oxidative stress markers showed a significant increase in nitrite and ROS levels, a decrease in non-oxidized GSH, and an increase in oxidized GSSG, resulting in an overall reduction in the GSH/GSSG ratio in the soman-vehicle group. SAR treatment significantly mitigated these effects, except for the GSH/GSSG ratio with a marginal recovery in GSH levels (Fig. 11a–e). Interestingly, the fold change in all serum oxidative markers were comparable, despite differences in their absolute values following soman exposure (Fig. 11f).

### SAR mitigated soman-induced proinflammatory cytokines in serum and CSF at 18 weeks post- exposure

**Figure 12:**
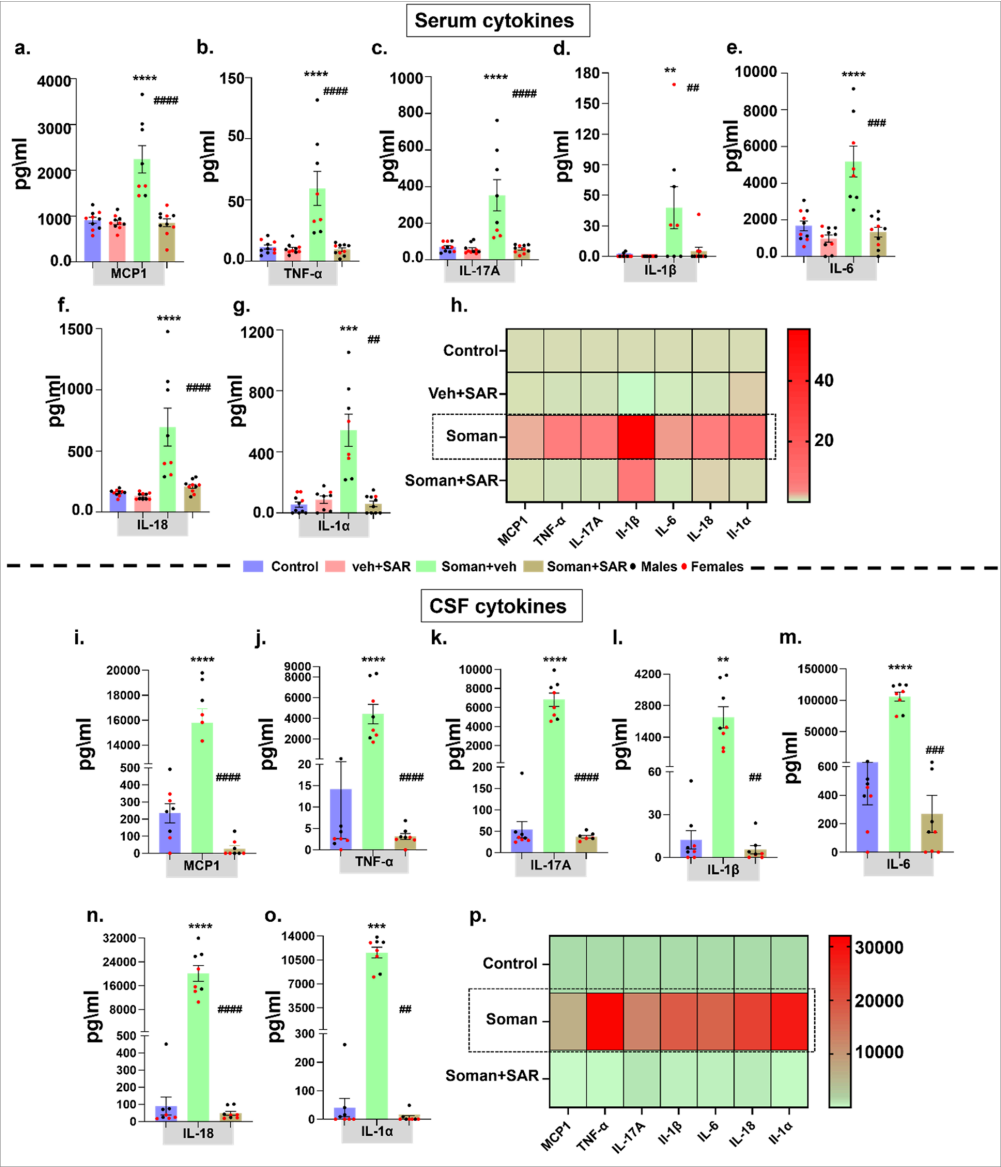
Secreted pro-inflammatory cytokine/chemokines in the serum and CSF of the rats at 18 weeks post-soman exposure. A customized MILLIPLEX^®^ Rat Cytokine/Chemokine kit was used to analyze cytokine/chemokine. The results are plotted as pg/mL for TNF-α, IL-6, IL-17A, MCP-1, IL-18, and IL-1α in (a-g) serum and CSF (i-o) and as (h & p) heatmaps to represent fold change, compared to the control (at 1), are displayed as a heatmap. Normality was assessed with the Shapiro-Wilk test. Stars indicate soman effect (soman vs control) and # represents SAR effect (soman+SAR vs Soman+vehicle). Bars represent mean ± SEM and each dot on the bar graphs represent a data point for each animal. n = 10 (5/sex). */^#^p < 0.05, **/^##^p < 0.01, ***/^###^p < 0.001, ****/^####^p < 0.0001 by two-way ANOVA with Tukey’s multiple comparisons. No sex differences were observed, therefore the data from both sexes were pooled.

Serum (Fig. 12a–g) and CSF (Fig. 12i–o) analysis revealed a significant rise in the levels of MCP1, TNF-α, IL-17A, IL-1β, IL-6, IL-18, and IL-1α following soman-exposure, and SAR treatment significantly mitigated the effect. Despite differences in absolute cytokine and chemokine levels, the fold change in serum were comparable across all cytokines upon soman exposure without SAR treatment (Fig. 12h). Similar trend in the fold change were observed in the CSF fraction of the soman exposed animals (Fig. 12p)

## Discussion

The primary objective of this study was to evaluate the therapeutic efficacy of SAR in mitigating soman-induced behavioral deficits, neuropathology, spontaneous seizures, and peripheral biomarkers such as nitro-oxidative stressors and proinflammatory cytokines. Given the critical involvement of Src family tyrosine kinases in neuroinflammation and neurodegeneration, we tested SAR as a therapeutic candidate targeting these pathological pathways (Nygaard et al. 2015; Sharma et al. 2021). Following acute exposure to soman, all animals developed severe *status epilepticus* lasting well over 25 minutes. Animals with comparable SE severity from both sexes were randomly assigned for vehicle or SAR treatment. Our previous findings from the DFP rat model highlight the critical role of SE severity, where seizures lasting over 20 minutes resulted in extensive neurodegeneration across multiple brain regions, while shorter seizures (< 20 minutes) caused localized changes in the piriform cortex and amygdala with significant gliosis and neurodegeneration (Massey et al. 2023). We have also confirmed that in addition to SE severity, the duration of SAR treatment is a critical factor in determining the neurological outcomes (Gage et al. 2021). In our previous rat kainate model, one-week SAR treatment (25mg/kg, oral gavage) twice a day for the first three days followed by a single dose in the next four days (1-week treatment) significantly reduced spontaneous seizures, neuroinflammation, and neurodegeneration in a four-month rat study (Sharma et al. 2021). In another rat kainate study, two weeks of SAR treatment (20mg/kg, oral) after the onset of first or second spontaneous seizures also reduced subsequent seizures in a 3-month continuous video-EEG study (Putra et al. 2024a). A two-week SAR regimen in a mouse kainate model improved memory and prevented neuronal loss (Liu et al. 2022). However, in a DFP rat model, short-term SAR treatment showed limited efficacy and higher concentrations of SAR were toxic in the DFP model implying reducing the dose may be therapeutically beneficial (Gage et al. 2021). Given that DFP is considered a surrogate for soman, in this study, an extended but tapering SAR dosing regimen over 18 weeks was tested to investigate its’ mitigating effect on soman-induced long-term neurotoxicity. A long-term and low-dose SAR treatment regimen in an Alzheimer’s disease mouse model showed that mice receiving SAR for 4 weeks demonstrated significant improvements in spatial memory (Smith et al. 2018). In this study, SAR administration began by oral gavage for the first week followed by SAR-in-diet with decreasing concentrations over time for up to 18 weeks. Our previous study has confirmed that this dosing regimen yield comparable serum and hippocampal drug concentrations and were well tolerated in the rat model (Vasanthi et al. 2023a). Additionally, the SAR-in-diet approach also minimizes handling stress while ensuring steady-state drug levels, aligning with the rat’s natural nocturnal feeding habits. Further supporting our dietary SAR strategy, similar dietary approach for other test drugs have been employed to effectively mitigate neuroinflammation and memory loss in animal models (Chaturvedi et al. 2021).

In this study, the open field and the elevated zero maze tests revealed anxiety-like behavior in soman-exposed animals. The elevated zero maze was designed to reduce potential confounding factors related to basic locomotor deficits that can be detected in the open field test. Additionally, the circular design of the elevated zero maze eliminates conflict zones, promoting continuous exploration while clearly distinguishing between open and closed areas. A decline in recognition, spatial and contextual memory was also seen in rats at 5 to 7 weeks after soman exposure. These impairments were consistent with the findings in other neurotoxic and neurodegeneration models, and from our previous study in soman model (Pentkowski et al. 2021; Vasanthi et al. 2023b). The limbic system (hippocampus, amygdala, hypothalamus, and thalamus), prefrontal cortex, the bed nucleus of the stria terminalis (BNST), and their functional connectivity network play a critical role in regulating anxiety/fear-like behaviors, together with learning and memory deficits (Seignourel et al. 2008; Martin et al. 2010). SAR treatment significantly mitigated anxiety-like behavior, likely through anti-inflammatory and antioxidant mechanisms that stabilized limbic neurocircuitry, thereby promoting recovery in emotional behavior.

This soman-induced hypersensitivity suggests disruption in somatosensory pathways, including the thalamus, medulla, and cerebral cortex (Wu et al. 2014). War veterans exposed to organophosphates warfare agents or traumatic brain injury may experience enhanced vulnerability to developing post-traumatic stress disorder (PTSD)-like symptoms, including hypervigilance and sensory hypersensitivity. Elevated proinflammatory cytokines in serum are known to induce hyperexcitability of peripheral sensory nerve terminals leading to hypersensitivity (Atta et al. 2023; Liu et al. 2019). While hypervigilance manifests as an exaggerated state of awareness and constant scanning for potential threats, hyperesthesia involves increased sensitivity to sensory stimuli such as touch or pain (Matsuda et al. 2019; Chen et al. 2018). Both conditions likely stem from dysregulated neural circuits within the limbic system and amplified inflammatory and autonomic responses, commonly triggered by chronic stress or traumatic experiences (Clancy et al. 2017; Katrinli et al. 2022).

While SAR effectively mitigated anxiety-like behavior and motor alterations, it failed to improve recognition, spatial, or contextual memory, suggesting its therapeutic effects at 5–7 weeks were more pronounced in lower-order neurological functions rather than higher cognitive processes governed by the prefrontal cortex and hippocampus (Frith and Dolan 1996; Nuss 2015). This distinction highlights SAR’s ability to regulate emotional and motor pathways, likely through anti-inflammatory and neuroprotective mechanisms, while being insufficient to restore complex memory functions for 5–7 weeks of treatment (the time the behavioral tests were conducted). Additionally, mitigating systemic inflammation and peripheral neuropathy may have contributed to reducing sensory hypersensitivity, as chronic neuroinflammation and peripheral nerve dysfunction are known to exacerbate autonomic dysregulation (Pitake et al. 2019). These findings suggest that while SAR provides partial neuroprotection after 5–7 weeks of treatment, targeting higher-order cognitive deficits may require longer SAR therapy to restore hippocampal and cortical connectivity, synaptic integrity, and excitatory/inhibitory balance. In this study, we did not repeat the behavioral tests at later time points.

MRI scans performed at 8–9 weeks post-exposure revealed a significant reduction in functional connectivity in the hippocampus. This alteration in the functional connectivity network was observed simultaneously with the behavioral deficits, notably impairments in cognitive performance, increased anxiety-like behavior, impaired fear extinction, and motor alterations (hyperesthesia). This association underscores the broad neurological impact of soman exposure on key neural circuits involved in emotional processing, memory, and motor control. SAR treatment significantly restored hippocampal connectivity at 8–9 weeks post-exposure (Fig. 6), a region known for regulating emotional and stress responses (Bannerman et al. 2004; Schoenfeld et al. 2016) .

Notably, by 18 weeks post-exposure, SAR treatment exhibited broader neuroprotective effects beyond the hippocampus, significantly mitigating neuronal degeneration across multiple brain regions, including the cortex and amygdala, except thalamus. Histological analysis demonstrated a marked reduction in Fluro-Jade B (FJB) positive degenerating neurons in SAR-treated animals compared to untreated controls, reflecting the extended benefits of prolonged SAR treatment. These later-stage improvements suggest that longer SAR therapy may progressively restore functional connectivity across additional limbic structures, potentially yielding more comprehensive cognitive recovery if treatment is maintained beyond the initial 8–9 weeks assessment period. Thus, isolated early improvement in hippocampal connectivity alone appears insufficient for full cognitive restoration, emphasizing the need for extended or combinational therapies targeting broader limbic and cortical networks to achieve meaningful functional recovery after soman-induced neurological injury (Bari et al. 2014; Tang et al. 2022).

During the nine weeks (60 days) of continuous video-EEG acquisition from weeks 10–18 post soman, the soman-vehicle group was maintained on the regular diet without SAR. Continuous video EEG (vEEG) data analysis revealed that SAR treatment modulated spontaneously recurring seizure characteristics, notably reducing the proportion of convulsive seizures (number and duration) compared to soman animals without SAR treatment. This modulation predominantly occurred after week 14 post soman, suggesting a progressive improvement in the excitatory/inhibitory (E/I) balance during this period. Interestingly, at 18 weeks post soman, brain sections analysis showed that SAR treatment restored somatostatin (SS) inhibitory neurons in the key brain regions investigated, except the motor cortex, while parvalbumin (PVB) inhibitory neurons remained significantly depleted except in the motor cortex (supplementary Fig. 4a-b). This selective restoration of inhibitory neuronal populations likely contributed to the observed shift toward milder seizures rather than reducing the overall seizure frequency or duration. PVB interneurons are the major subtypes of inhibitory GABAergic neurons that constitute about 50% in the cortex and 30% in the hippocampus (Wonders and Anderson 2006; Bezaire and Soltesz 2013; Kosaka et al. 1987). PVB interneurons generate fast-spiking action potential that allow for very efficient communication within neuronal networks and generate gamma oscillations (Bartos et al. 2007; Cardin et al. 2009; Sohal et al. 2009). These gamma rhythms are crucial for regulating perception, attention, and memory (Uhlhaas and Singer 2010). Loss or dysregulation of PVB interneurons can lead to epileptiform spikes, seizures, and anxiety-like disorders (Wonders and Anderson 2006; Buckmaster et al. 2017).

In this study, the persistent epileptiform spiking and seizures could be due to a significant loss of PVB neurons. The persistent epileptiform spiking activity despite SAR treatment could thus reflect an ongoing synaptic plasticity reorganization between the survived inhibitory neurons and possibly due to delayed recovery of PVB and SS inhibitory neurons (Călin et al. 2021). These findings align with the literature (Leitch 2024) emphasizing the distinct roles of inhibitory neuron subpopulations in modulating seizure dynamics and suggest that therapies targeting specific inhibitory circuits could substantially influence seizure severity and clinical outcomes. For example, in a similar rat soman model, 1400W treatment for two weeks post-exposure significantly rescued PVB interneurons in the amygdala and there was significant reduction in spontaneous seizures and epileptiform spikes, which could not be achieved with long term SAR treatment implying the neuroprotective mechanism could be unique to some subpopulations of inhibitory neurons (Vasanthi et al. 2023b). Further studies exploring such mechanisms or combination treatments may restore specific inhibitory pathways and control seizures in long-term (Bravo et al. 2021; Liu et al. 2020; van van Hugte et al. 2023).

Glial scarring is a hallmark response to severe neuronal injury and neurodegeneration, characterized by robust astrocytic activation and extracellular matrix (ECM) deposition (Bradbury and Burnside 2019). Consistent with this, prominent glial scars were observed in the amygdala of soman-exposed animals, highlighting significant localized neurodegeneration and persistent inflammatory responses following acute soman exposure. The formation of these glial scars likely contributed to chronic functional impairments by impeding neuronal connectivity and synaptic plasticity within critical limbic pathways involved in emotional processing and memory. SAR treatment significantly reduced glial scar formation (Fig. 9). Quantitative analysis indicated a significant decrease in glial scar-positive sections and total scar volume in SAR-treated animals compared to the soman only group. The observed reduction in scarring likely resulted from SAR’s ability to mitigate reactive gliosis and inflammation, processes critical in the early stages of scar development (Vasanthi et al. 2023b). Consistent with findings from chemical induced epilepsy models, the glial scars in our model featured reactive astrocytes forming a distinct peripheral ring with a central core filled with CD68 containing microglia and few degenerating neurons (Rao et al. 2023). The glial scar morphology also aligns closely with previous reports in the DFP model, where chondroitin sulfate proteoglycans (CSPGs) were predominantly expressed in reactive astrocytes surrounding glial scars, highlighting the importance of ECM components in scar formation (Gage et al. 2022). While CSPGs play a substantial role, earlier studies suggested variable involvement of Transforming growth factor-β (TGF-β) signaling depending on the timing and nature of chemical insult (Yang et al. 2020; Bradbury and Burnside 2019). However, in our previous DFP study, no change in TGF-β1 or TGF-β2 positive cells in or around the scars in DFP-exposed animals compared to controls (Gage et al. 2022). Our findings in this study underscore SAR’s efficacy in reducing glial scar formation, potentially through targeted suppression of reactive astrogliosis. Additionally, the distinctive vulnerability of the amygdala and piriform cortex to seizure-induced damage may further enhance persistent scarring independent of SAR’s primary therapeutic mechanisms (Malkova et al. 1997).

Reactive astrocytes (GFAP + C3) and reactive microglia (IBA1 + CD68) promote seizure dynamics and epileptogenesis through complex inflammatory mechanisms (Sano et al. 2021). Brain histological analyses at 18 weeks post-soman exposure demonstrated significant reactive gliosis, characterized by increased numbers of activated microglia and reactive astroglia across the hippocampus, cortex, thalamus, and amygdala. SAR treatment effectively mitigated reactive gliosis, significantly reducing both astroglial and microglial activation within these critical brain regions. A similar outcome was observed in the rat kainate model treated with SAR (Sharma et al. 2021). By attenuating glial-driven inflammatory responses, SAR likely limits the release of pro-inflammatory cytokines and chemokines and reduces oxidative stress (Figs. 11&12). The observed cellular and peripheral biomarkers improvements support that the prolonged SAR treatment.

Microglia are the resident sensors of the CNS microenvironment, rapidly detect neuronal stress or damage and regulate astrocyte activity via intricate microglia-astrocyte crosstalk mediated by C3 and C1q interaction (Vandenbark et al. 2021). These cells actively produce proinflammatory cytokines and chemokines, enhancing inflammatory signaling within the CNS and driving chronic neuronal excitability (Vezzani and Viviani 2015). Astrocytes, with their close physical associations to neurons and blood vessels, further amplify these inflammatory responses, sensing neuronal damage and modulating microglial activation to sustain chronic neuroinflammation (Minkiewicz et al. 2013; Tapia-Abellán et al. 2021). Specifically, reactive astrocytes release chemokines such as MCP1, which binds to CCR2 receptors expressed on stressed neurons and activated microglia, perpetuating a pro-inflammatory cycle that exacerbates neuronal hyperexcitability (Tanuma et al. 2006).

A prior study showed that in brain tissue, certain glial cells (identified by IBA1) increased iNOS production, leading to the creation of reactive nitrogen species (RNS). These RNS reactive results in nitrosylation of neuronal proteins (3NT) (Putra et al. 2020). These nitro-oxidative processes can contribute to neurodegeneration, altered excitability, and increased seizure susceptibility, ultimately promoting the initiation and propagation of epileptic seizures (Sharma et al. 2019). At 18 weeks post-soman exposure, SAR revealed pronounced antioxidant and anti-inflammatory properties, significantly reducing markers of oxidative stress, such as improving the GSH/GSSG ratio and lowering levels of pro-inflammatory cytokines and chemokines including MCP-1, TNF-α, IL-6, IL-1β, IL-18, and IL-17A. These biochemical parameter improvements closely align with our histological findings, highlighting SAR’s ability to effectively suppress systemic inflammatory responses and oxidative injury critical processes known to drive chronic neurodegeneration after soman exposure. Thus, SAR’s anti-inflammatory and antioxidant mechanisms likely play a central role in preventing secondary neuronal damage.

A notable limitation of this study is the timing of different assessments. Behavioral studies and MRI imaging were performed at 5–7 and 8–9 weeks post-exposure, respectively, while histological analysis from 18 weeks post-exposure. This time gap between studies potentially led to discrepancies in observed neuroprotective effects versus behavioral outcomes. Collectively, our findings suggest that prolonged SAR administration in descending concentrations provides substantial neuroprotective effects through targeted suppression of inflammation, oxidative stress, and selective preservation of inhibitory neuronal circuits. Future studies extending treatment duration or employing combinatorial therapeutic approaches such as 1400W, which rescued parvalbumin interneurons in contrast to SAR, or with antiseizure medication for long-term could further enhance inhibitory neurons restoration, potentially resulting in broader functional recovery and further improved outcomes after nerve agent exposure.

## Conclusion

This study provides compelling evidence that prolonged SAR administration offers neuroprotective and disease modifying effects in a rat model of soman-induced neurotoxicity. SAR significantly reduced seizure severity, mitigated anxiety-like behavior and motor impairments, and partially preserved inhibitory interneurons. While in the short term, SAR treatment did not reverse cognitive deficits, long-term administration markedly attenuated neurodegeneration, glial scar formation, and reactive gliosis across key brain regions. In parallel, SAR reduced systemic nitro-oxidative stress and proinflammatory cytokine levels, underscoring its anti-inflammatory and antioxidant mechanisms of action. These findings highlight Src kinase inhibition as a promising adjunct strategy to mitigate chronic neurological sequelae following OPNA exposure. By disrupting the interconnected cascade of seizures, neuroinflammation, structural damage, and neurobehavioral deficits, SAR demonstrates translational potential to address critical gaps in current medical countermeasures. This work advances our understanding of long-term neuroprotection and provides a strong foundation for future studies involving combination therapies or extended SAR therapies for chemically induced epileptogenesis and neurodegeneration.

## Supplementary Material

Supplementary Files

This is a list of supplementary les associated with this preprint. Click to download.

• SupplementaryFile.docx

## Figures and Tables

**Table 1:** Experimental groups details

Treatment groups	Treatment	Number of animals	Mortality	SE severity (Mean ± SEM)
Control	Cold PBS (i.m.; once) + HPMC, oral gavage (week 0)	25 (13 females/12 males)	0	NA
veh + SAR	Cold PBS (i.m.; once) + SAR oral gavage (week 0) and in-diet (18 weeks)	25 (12 females/13 males)	0	NA
Soman + veh	Soman (s.c., once) + HPMC, oral gavage (week 0)	25 (12 females/13 males)	4 (3 females, 1 male)	51.84 ± 1.08
Soman + SAR	Soman (s.c., once) + SAR oral gavage (week 0) and in-diet (18 weeks)	25 (13 females/12 males)	8 (4 females, 4 male)	52 ± 1.17

**Table 2:** Summary of biological replicates and sample type for individual analysis

Analysis	Sample type	Number of animals
Neurobehavioral tests	Live animals	22–25 (10–13/sex)
MRI analysis	Live animals	8–16 (4–8/sex)
EEG analysis	Live animals	8–12 (3–6/sex)
LC/MS analysis (serum)	Serum	9–10 (4–5/sex)
Secreted cytokines	Serum	10 (5/sex)
Secreted free radicals	Serum	10 (5/sex)
Immunohistochemistry	Perfused tissue	10 (5/sex)

**Table 3:** SAR-in-diet feeding regimen

Estimated PPM	Analyzed PPM (LC/MS)	Targeted dose (mg/kg)	Actual dose received (mg/kg)	# of weeks fed
260	260.15	15–20	17.47	1
210	219.41	10–15	13.75	1
160	165.45	5–10	8.95	5
50	54.43	2.5–5	2.75	11

**Table 4:** Antibodies used for IHC

Primary Antibody	Source	Catalogue number
Anti-IBA1	Abcam	ab5076
Anti-CD68	Abcam	Ab125212
Anti-GFAP	Sigma Aldrich	G3893
Anti-C3	Novus Biologicals	NM200540
Anti-NeuN	EMD Millipore	ABN78
Anti-Parvalbumin	Abcam	Ab11427
Anti-Somatostatin	EMD Millipore	AB5494
Secondary Antibody	Source	Catalogue number
AMCA blue streptavidin	Jackson ImmunoResearch	016-150-084
Biotinylated Donkey anti-Goat	Jackson ImmunoResearch	711-295-152
AlexaFluor 488 anti-mouse	Jackson ImmunoResearch	705-065-147
Rhodamine Red X anti-rabbit	Jackson ImmunoResearch	715-545-150

## Data Availability

The datasets used and/or analyzed during the current study are available from the corresponding author on reasonable request and can be accessed as supplementary information files.

## Abbreviations

AChE: Acetylcholinesterase
ANOVA: Analysis of Variance
ATS: Atropine Sulfate Monohydrate
BOLD: Blood Oxygenation Level Dependent
CA1/CA3: Cornu Ammonis areas 1 and 3 (hippocampal subfields)
CCR2: C-C Motif Chemokine Receptor 2
CNS: Central Nervous System
CSF: Cerebrospinal Fluid
CSPGs: Chondroitin Sulfate Proteoglycans
DAPI: 4′6-Diamidino-2-Phenylindole
DG: Dentate Gyrus
DFP: Diisopropylfluorophosphate
ECM: Extracellular Matrix
EEG: Electroencephalography
E/I: Excitatory/Inhibitory (balance)
FIESTA: Fast Imaging Employing Steady-State Acquisition
FJB: Fluoro-Jade B
GFAP: Glial Fibrillary Acidic Protein
GSH: Reduced Glutathione
GSSG: Oxidized Glutathione
HI-6: Asoxime chloride (oxime compound)
HPMC: Hydroxypropyl Methylcellulose
IBA1: Ionized Calcium-Binding Adapter Molecule 1
i.m.: Intramuscular
i.p.: Intraperitoneal
IHC: Immunohistochemistry
IL: Interleukin
iNOS: Inducible Nitric Oxide Synthase
LD50: Median Lethal Dose
LC-MS/MS: Liquid Chromatography–Mass Spectrometry/Mass Spectrometry
LLOQ: Lower Limit of Quantification
MCM: Medical Countermeasure
MCP-1: Monocyte Chemoattractant Protein 1
MDZ: Midazolam
MRI: Magnetic Resonance Imaging
OPNA: Organophosphate Nerve Agent
PFA: Paraformaldehyde
PBS: Phosphate-Buffered Saline
ppm: Parts Per Million
PVB: Parvalbumin
RFU: Relative Fluorescence Units
RNS: Reactive Nitrogen Species
ROS: Reactive Oxygen Species
RSFC: Resting-State Functional Connectivity
SAR: Saracatinib (AZD0530)
s.c.: Subcutaneous
SE: Status Epilepticus
SFK: Src Family Kinase
SRS: Spontaneously Recurring Seizures
SS: Somatostatin
vEEG: Video Electroencephalography
